# Multimodal AI in Biomedicine: Pioneering the Future of Biomaterials, Diagnostics, and Personalized Healthcare

**DOI:** 10.3390/nano15120895

**Published:** 2025-06-10

**Authors:** Nargish Parvin, Sang Woo Joo, Jae Hak Jung, Tapas K. Mandal

**Affiliations:** 1 School of Mechanical Engineering, Yeungnam University, Gyeongsan 38541, Republic of Korea; nargish.parvin@gmail.com (N.P.); swjoo1@gmail.com (S.W.J.); 2 School of Chemical Engineering, Yeungnam University, Gyeongsan 38541, Republic of Korea

**Keywords:** multimodal AI, biomaterials science, AlphaFold, wearable healthcare, regenerative medicine

## Abstract

Multimodal artificial intelligence (AI) is driving a paradigm shift in modern biomedicine by seamlessly integrating heterogeneous data sources such as medical imaging, genomic information, and electronic health records. This review explores the transformative impact of multimodal AI across three pivotal areas: biomaterials science, medical diagnostics, and personalized medicine. In the realm of biomaterials, AI facilitates the design of patient-specific solutions tailored for tissue engineering, drug delivery, and regenerative therapies. Advanced tools like AlphaFold have significantly improved protein structure prediction, enabling the creation of biomaterials with enhanced biological compatibility. In diagnostics, AI systems synthesize multimodal inputs combining imaging, molecular markers, and clinical data—to improve diagnostic precision and support early disease detection. For precision medicine, AI integrates data from wearable technologies, continuous monitoring systems, and individualized health profiles to inform targeted therapeutic strategies. Despite its promise, the integration of AI into clinical practice presents challenges such as ensuring data security, meeting regulatory standards, and promoting algorithmic transparency. Addressing ethical issues including bias and equitable access remains critical. Nonetheless, the convergence of AI and biotechnology continues to shape a future where healthcare is more predictive, personalized, and responsive.

## 1. Introduction

### 1.1. Overview of Multimodal AI

Multimodal artificial intelligence (AI) is an emerging and transformative domain that combines multiple data modalities to enhance decision-making, especially in intricate fields such as healthcare and biomedical research. Unlike traditional AI systems that analyze a single data stream, like medical images or textual data, multimodal AI integrates diverse sources such as clinical imaging, genetic profiles, biosensor outputs, and electronic health records. This integrative approach enables a deeper and more unified interpretation of human biology and disease. In the context of healthcare, where the data variety is immense and complexity is high, multimodal AI offers unprecedented potential. It effectively synthesizes information from numerous biomedical sources, including high-resolution scans, genomic sequences, patient histories, and continuous physiological monitoring from wearable devices, thereby enabling more accurate and personalized medical insights [1]. Each data type, on its own, provides valuable insights into a patient’s health. However, combining these datasets allows researchers and clinicians to uncover complex relationships between physiological and genetic factors, leading to more accurate diagnoses, personalized treatments, and improved outcomes [2]. Multimodal AI systems rely on advanced machine learning techniques, particularly deep learning, which can process large, complex datasets and identify patterns that would be impossible for humans to detect. For example, convolutional neural networks (CNNs) are used for analyzing medical images, while recurrent neural networks (RNNs) process time-series data such as electrocardiograms (ECGs) [3]. CNNs are advanced deep learning models specifically effective for analyzing visual data. By applying layered filters, these networks can autonomously recognize and extract intricate visual features such as edges, contours, and textures. This makes them highly suitable for medical diagnostics, where they are used to identify irregularities such as tumors in imaging techniques like MRIs and X-rays. RNNs are a distinct type of neural architecture tailored for sequential information. They excel in tasks involving temporal patterns due to their internal memory, which allows them to utilize previous inputs for current predictions. In healthcare, RNNs have been leveraged to analyze longitudinal patient data, helping forecast disease development or clinical outcomes over time. By combining diverse data sources, multimodal AI enables in-depth assessments that support early disease detection, personalized therapy recommendations, and continuous monitoring especially beneficial for managing chronic illnesses like cardiovascular disorders and diabetes [4]. Convolutional neural networks (CNNs) are frequently employed to interpret and merge various imaging techniques, such as MRI, CT, and X-ray, offering more holistic diagnostic insights. These models can be integrated with patient clinical data to assist in designing individualized treatment strategies. Meanwhile, recurrent neural networks (RNNs) are commonly applied in genomic studies to analyze DNA or RNA sequences. These models help identify genetic variations and forecast their impact on disease mechanisms, facilitating precision medicine and the development of targeted therapies.

### 1.2. Importance of AI in Biomedicine and Healthcare

The application of AI, especially multimodal AI, in biomedicine and healthcare is increasingly viewed as a game changer. Traditionally, healthcare systems have struggled to integrate different forms of patient data, which has led to challenges in diagnosing complex diseases, tailoring personalized treatments, and predicting outcomes. Multimodal AI overcomes traditional limitations by synthesizing data from multiple sources to construct a holistic representation of patient health. This comprehensive approach supports more accurate diagnoses and paves the way for predictive and individualized treatments [5]. A key area where multimodal AI is proving transformative is in the field of biomaterials. These materials whether synthetic or natural are engineered to interface with biological systems for medical applications such as regenerative therapies, drug delivery platforms, and tissue scaffolding [6]. The development of such materials involves evaluating a wide range of parameters, including biomechanical performance, biocompatibility, and patient-specific physiological factors. Multimodal AI enables the integration and analysis of these complex datasets, allowing for the design of highly tailored and functionally superior biomaterials [7].

AI-driven systems can synthesize diverse datasets, such as mechanical properties from materials engineering, biological responses from tissue studies, and clinical information from patient records, to develop advanced biomaterials for tissue regeneration. This integrative approach has contributed to the creation of innovative scaffolds and hydrogels that better replicate the structural and functional characteristics of native tissues, thereby enhancing the efficacy of regenerative therapies [8,9]. In addition to biomaterials, multimodal AI is transforming diagnostics and personalized medicine by merging medical images, genomic profiles, and clinical histories. Such fusion enables the predictive modeling of treatment outcomes, facilitating the selection of the most effective interventions for individual patients. This is especially impactful in cancer care, where therapeutic decisions like chemotherapy regimens can be guided by tumor genomics and radiological imaging [10,11]. Moreover, real-time health surveillance is being revolutionized through the integration of multimodal AI with wearable health technologies. Devices like fitness trackers and smartwatches continuously capture vital signs such as heart rate, body temperature, and physical activity. When combined with clinical and genomic data, this stream of information allows AI systems to deliver dynamic health assessments, early disease detection, and customized lifestyle guidance [12]. These advancements support a shift toward preventative medicine, enabling earlier interventions and contributing to reduced healthcare expenditures while improving patient outcomes [13].

### 1.3. Objectives of the Review

This review aims to elucidate the transformative role of multimodal artificial intelligence (AI) in reshaping the landscape of modern biomedicine, with a specific focus on biomaterials engineering, diagnostic innovations, and precision medicine. By synthesizing diverse data modalities, ranging from medical imaging and genomics to clinical and physiological data, multimodal AI offers novel avenues to enhance patient care, streamline clinical workflows, and accelerate biomedical innovation. This article provides a detailed examination of how AI technologies are being applied to facilitate data-driven decision-making and optimize biomedical interventions. In addition, we address the practical challenges associated with the clinical adoption of AI, including ethical dilemmas, regulatory constraints, data standardization, and implementation at scale [14]. Looking forward, we explore the convergence of multimodal AI with frontier biotechnologies, such as CRISPR-based gene editing and tailored therapeutics, underscoring its potential to redefine the standards of healthcare delivery [15]. Ultimately, the integration of heterogeneous biomedical data through AI holds the promise to propel the field toward more proactive, predictive, and personalized medical solutions.

### 1.4. Materials and Methods

#### 1.4.1. Databases Searched

We conducted a comprehensive literature search using the following databases to cover interdisciplinary research spanning artificial intelligence, biomaterials, and healthcare:

- **PubMed:** For peer-reviewed articles on biomedical applications of AI, biomaterials development, and clinical healthcare innovations.
- **IEEE Xplore:** For technical research on AI algorithms such as convolutional neural networks (CNNs), recurrent neural networks (RNNs), and their deployment in biomedicine.
- **Scopus:** For a wide spectrum of research literature in materials science, biomedical engineering, and AI applications.
- **Web of Science:** For studies on the integration of AI technologies in healthcare and biomedical research.

#### 1.4.2. Materials and Methods for AI Model Development

In addition to literature sources, we reviewed datasets and resources essential for the development and training of AI models relevant to biomaterials and healthcare:

- **Protein Data Bank (PDB):** A critical resource for structural biology, used in training deep learning models like AlphaFold for accurate protein structure prediction. PDB provides experimentally validated protein and macromolecular structures, forming the backbone of structure-based AI applications.
- **Medical Imaging Datasets:** Publicly available databases such as The Cancer Imaging Archive (TCIA) and NIH Chest X-ray dataset were considered for studies utilizing AI in diagnostic imaging.
- **Electronic Health Records (EHRs):** Studies incorporating AI in patient stratification and clinical decision-making often utilized anonymized EHR datasets like MIMIC-III.
- **Genomic Databases:** Sources such as The Cancer Genome Atlas (TCGA) were used in multimodal AI models combining genetic, imaging, and clinical data.

## 2. Role of Multimodal AI in Biomaterials Development

The integration of multimodal artificial intelligence (AI) into biomaterials development represents a transformative advancement in the biomedical sciences. Biomaterials comprising both natural and synthetic materials engineered for interactions with biological systems are fundamental to applications such as tissue engineering, drug delivery, and regenerative therapies. Historically, the design and optimization of these materials have relied heavily on trial-and-error methodologies, wherein individual materials are experimentally evaluated for attributes like biocompatibility, mechanical strength, and degradation behavior. This conventional process is often time-consuming, resource-intensive, and limited in scope.

However, this process can be slow and resource-intensive. Multimodal AI, which integrates data from various sources, like medical imaging, genomic data, and clinical information, is revolutionizing this process by accelerating material discovery, optimizing biomaterial design, and improving patient-specific applications [16,17]. Table 1 illustrates how multimodal AI is enhancing biomaterials development, offering an improved personalization, prediction accuracy, and development speed while highlighting new ethical and regulatory considerations [18,19,20,21,22,23,24,25,26].

### 2.1. Combining Multimodal Data (Imaging, Genomic, and Clinical Data)

A key aspect of multimodal AI in biomaterials research is the fusion of varied data types, including imaging, genomic, and clinical datasets. By analyzing the relationships between biological, mechanical, and patient-specific information, researchers gain a deeper understanding of how biomaterials perform in real-world applications. For instance, in tissue engineering, optimal material design requires balancing mechanical compatibility with host tissues while also ensuring the material supports cell adhesion, proliferation, and seamless biological integration [27]. This holistic approach enhances the development of biomaterials tailored for specific medical needs.

#### Leveraging Medical Imaging for Biomaterial Design

Advanced imaging techniques including MRI, CT scans, and high-resolution microscopy provide critical insights for biomaterial development. These methods enable a detailed visualization of tissue morphology, mechanical characteristics, and microstructural patterns, which are vital for designing functional tissue scaffolds. AI-driven approaches, especially convolutional neural networks (CNNs), excel at analyzing intricate imaging datasets, identifying key features such as tissue porosity, fiber alignment, and biomechanical properties. By automating feature extraction, AI enhances the precision of biomaterial optimization for regenerative medicine and tissue engineering applications [28]. By analyzing these features, multimodal AI systems can design materials with tailored mechanical properties that closely mimic those of native tissues. For instance, in cartilage tissue engineering, it is critical to design scaffolds that mimic the anisotropic mechanical properties of cartilage, such as its ability to withstand compressive forces. The AI-driven analysis of MRI scans of cartilage can help identify these properties, guiding the design of materials with comparable characteristics [29]. Similarly, in bone tissue engineering, CT scans provide detailed information about the microarchitecture of bone, which can be used to develop materials with an optimized porosity and strength.

### 2.2. Harnessing Genomic Data for Personalized Biomaterials

The incorporation of genomic data into biomaterial design enables a precision medicine approach, optimizing compatibility and therapeutic efficacy. By analyzing individual genetic profiles, multimodal AI can predict how a patient’s immune system may react to an implant—identifying risks such as inflammation or rejection due to specific mutations or polymorphisms. Machine learning models can then recommend biomaterials that minimize adverse immune responses while promoting integration. In regenerative medicine, an AI-driven genomic analysis plays a pivotal role in material selection for stem cell therapies. By correlating genetic markers with cellular behavior, algorithms can determine which biomaterials best support differentiation and tissue regeneration. For instance, certain scaffold compositions may be prioritized based on a patient’s gene expression patterns to enhance osteogenesis or angiogenesis. This tailored strategy not only improves clinical outcomes but also mitigates potential complications, advancing the frontier of patient-specific therapeutic solutions [30,31].

#### Clinical Data Integration for Patient-Centric Biomaterial Design

**Figure 1:**
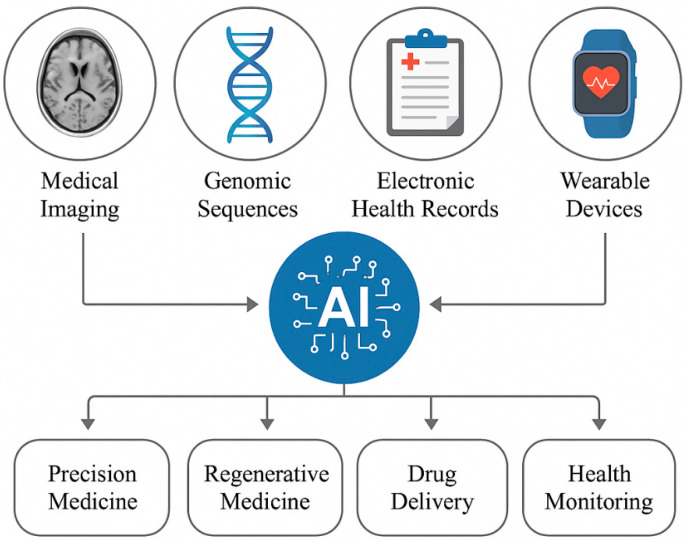
Various data modalities and potential applications of multimodal AI in biomedicine.

The incorporation of clinical data encompassing electronic health records, laboratory findings, and treatment responses provides a crucial context for optimizing biomaterial performance. By synthesizing this information with imaging and genomic datasets, multimodal AI enables the development of biomaterials that address both biological compatibility and individual clinical requirements. For instance, AI can evaluate comorbidities like diabetes or cardiovascular conditions to guide material selection for applications such as diabetic wound dressings or vascular implants, where healing dynamics may be significantly altered [32]. A particularly impactful application lies in smart drug delivery systems. AI models can process patient-specific variables including metabolic profiles, medication history, and treatment adherence to engineer biomaterials with tailored drug release kinetics. Such systems could adapt to individual pharmacokinetics, enhancing therapeutic efficacy while minimizing adverse effects. In oncology, for example, this approach might optimize chemotherapy-loaded scaffolds based on tumor responsiveness and patient tolerance [33]. The convergence of clinical analytics with biomaterial science thus represents a transformative step toward truly personalized medical solutions. By integrating these diverse data types, multimodal AI enables a holistic approach to biomaterials development, allowing researchers to design materials that are both biologically and mechanically suited to individual patients. Figure 1 illustrates the diverse data modalities that multimodal AI can integrate for biomedical applications, highlighting the wide-ranging opportunities this approach offers in advancing healthcare.

Modern healthcare leverages diverse data streams including medical imaging (MRI and CT), genomic profiles (DNA sequencing and transcriptomics), clinical records (EHRs and laboratory diagnostics), and wearable-derived physiological metrics to drive innovation in personalized medicine [34]. Multimodal AI synthesizes these heterogeneous datasets, revealing intricate correlations between genetic predispositions, structural abnormalities from imaging, and clinical manifestations. Such integration enhances diagnostic accuracy, enables dynamic treatment adaptations, and supports the predictive modeling of health trajectories.

This synergistic approach proves particularly transformative in three key areas:

Targeted Therapeutics: The AI-driven analysis of drug response patterns across multi-omics and clinical data informs precision drug formulation and delivery systems.

Tissue Engineering: Correlating mechanical properties from imaging with cellular behavior from genomic data facilitates scaffold customization for regenerative applications.

Proactive Care: Continuous wearable data combined with episodic clinical measurements enables early interventions through predictive analytics.

### 2.3. AI-Driven Biomaterial Design for Tissue Engineering

Traditional biomaterial development for tissue engineering has long depended on iterative experimental processes, involving extensive synthesis, characterization, and testing cycles. While these empirical methods have yielded valuable materials, they often prove resource-intensive and inefficient due to their reliance on trial-and-error optimization. The emergence of artificial intelligence is fundamentally reshaping this paradigm by introducing predictive, data-centric strategies that accelerate material discovery and refinement.

A critical application lies in tissue scaffold engineering, where three-dimensional architectures must precisely replicate native tissue characteristics. Key mechanical parameters including the elastic modulus, pore topology, and viscoelastic behavior require careful tuning to

➢ Promote cellular infiltration and nutrient diffusion through optimized porosity;
➢ Mimic tissue-specific mechanics (e.g., neural vs. musculoskeletal targets);
➢ Maintain structural integrity during dynamic remodeling processes.

AI models leverage existing experimental datasets to establish structure–property relationships, enabling an inverse design of scaffolds with predetermined performance criteria. Machine learning algorithms can predict how variations in the polymer composition, fabrication parameters, and architectural features will influence both the mechanical behavior and biological response [35]. This computational approach not only reduces development timelines but also facilitates the creation of graded or stimuli-responsive materials that adapt to evolving regenerative needs. An AI-enhanced scaffold design leverages medical imaging to create biomaterials that precisely mimic the native tissue architecture and biomechanics. Machine learning algorithms analyze CT/MRI datasets to identify critical structural patterns, enabling the data-driven fabrication of optimized tissue scaffolds. These features can then be used to guide the design of biomaterials with comparable properties. In the case of bone tissue engineering, AI can analyze CT scan data to determine the optimal porosity and strength required for scaffolds to support bone regeneration [36]. In cutaneous regeneration, artificial intelligence facilitates scaffold development by decoding skin microstructural patterns to enhance healing outcomes. Machine learning models evaluate material performance by synthesizing biological data, enabling the selection of substrates with optimal biocompatibility, cellular interaction properties, and controlled degradation kinetics [37]. A notable application involves hydrogel optimization, where AI cross-references imaging, molecular, and clinical datasets to determine formulations that best replicate natural extracellular matrix characteristics, thereby improving the regenerative potential [38]. Additionally, AI can guide the customization of these materials based on patient-specific factors, such as the location and severity of the injury. One of the most significant challenges in biomaterials development is predicting how materials will interact with cells and tissues once implanted. Multimodal AI offers a solution by enabling predictive modeling based on data from multiple sources, including in vitro experiments, imaging data, and clinical outcomes [39]. AI models can analyze these data to predict how cells will behave on different materials, including their adhesion, proliferation, and differentiation. For instance, in cartilage tissue engineering, AI-driven models can predict how chondrocytes will interact with different biomaterials, enabling the design of scaffolds that promote cartilage regeneration. Similarly, in vascular tissue engineering, AI can predict how endothelial cells will respond to specific materials, guiding the development of vascular grafts that promote blood vessel formation and reduce the risk of thrombosis [40]. Here Table 2 highlights how AI-driven approaches are revolutionizing biomaterial design by enabling data-driven optimization and patient-specific customization. Machine learning and deep learning techniques enhance scaffold properties (e.g., porosity and stiffness), while predictive modeling improves cell–material interactions for targeted tissue regeneration. The integration of multimodal data (imaging and genomics) underscores AI’s transformative role in developing precision biomaterials for diverse clinical applications, from bone repair to vascular grafts. Multimodal AI enables groundbreaking advances in patient-specific biomaterial designs by synthesizing individual imaging, genomic, and clinical profiles [41]. This precision approach proves particularly transformative for regenerative therapies, where the material–tissue compatibility directly determines therapeutic success. Sophisticated algorithms can predict individual immune reactions from genetic markers, informing the development of immune-compatible materials that reduce rejection risks. Furthermore, AI tailors biomechanical characteristics from bone scaffold rigidity to cutaneous graft flexibility to match each patient’s unique physiological requirements [42].

### 2.4. AI in Drug Delivery Systems

Multimodal artificial intelligence (AI) is revolutionizing drug delivery systems by integrating diverse datasets, such as clinical records, imaging data, and molecular characteristics, to create more precise and effective therapies. Drug delivery systems are critical in medicine, ensuring that therapeutic agents reach their intended targets in the body in the right quantities and at the right times. Conventional drug delivery systems often suffer from inefficiencies such as poor targeting, low bioavailability, and side effects. AI can optimize these systems by predicting and modeling how different drugs interact with biological environments, leading to personalized and targeted delivery approaches [43]. Nanoparticles serve as effective drug carriers by enhancing therapeutic solubility, stability, and bioavailability. However, optimizing their design remains challenging due to multiple interdependent parameters including size, morphology, surface chemistry, and material properties [44]. Multimodal AI accelerates this optimization by synthesizing diverse datasets from clinical studies, computational simulations, and imaging analyses to predict ideal nanoparticle configurations for targeted drug delivery. For example, AI can analyze high-throughput screening data from in vitro experiments to determine how the nanoparticle size and surface chemistry affect drug release kinetics and cellular uptake. Similarly, AI models can process imaging data from animal studies to predict how nanoparticles distribute throughout the body and accumulate at specific disease sites, such as tumors. This data-driven approach enables the design of nanoparticles that are more efficient at delivering drugs to their targets while minimizing off-target effects [45]. In a study, machine learning algorithms were applied to predict the bio-distribution and pharmacokinetics of nanoparticles in drug delivery applications. By integrating imaging data and pharmacological data, the AI model could optimize the nanoparticle design, improving both the targeting efficiency and safety profile of the drug delivery system [46].

By harnessing multimodal artificial intelligence, therapeutic delivery systems can now be precisely customized to match each patient’s distinct physiological profile. This AI-powered approach to individualized treatment represents a transformative advancement in modern healthcare, positioning smart drug delivery technologies as pivotal components in the emerging era of precision medicine. By integrating genomic, proteomic, and clinical data, AI models can predict how individual patients will respond to specific drugs, allowing for the design of delivery systems that maximize therapeutic efficacy while minimizing adverse effects [47]. One area where personalized drug delivery is particularly valuable is in cancer treatment. Tumors are highly heterogeneous, meaning that different patients with the same type of cancer may respond differently to the same drug. AI can integrate genomic data from tumor biopsies with imaging data and clinical histories to identify the most appropriate drug and delivery system for each patient [48]. For example, AI could analyze a patient’s genetic mutations and tumor microenvironment to select nanoparticles that will effectively penetrate the tumor and release the drug at the optimal rate. This level of precision could significantly improve outcomes for cancer patients, reducing the need for trial-and-error approaches to treatment [49]. Table 3 presents a comparative analysis of various AI-driven diagnostic models in biomedicine, highlighting their methodologies, accuracy, advantages, and limitations. Convolutional neural networks (CNNs) and recurrent neural networks (RNNs) demonstrate a superior performance in medical imaging and the sequential data analysis, respectively, with CNNs excelling in tumor detection and classification, while RNNs show promise in processing genomic and electronic health record data. Transformer-based models, such as attention mechanisms in multimodal AI, have enhanced diagnostic precision by integrating heterogeneous data sources [50,51,52,53,54].

Despite achieving impressive accuracy rates, AI diagnostic systems still face significant hurdles related to data quality requirements, algorithmic transparency, and inherent biases in training datasets. While conventional machine learning approaches like Support Vector Machines (SVMs) and decision trees provide more interpretable and computationally lightweight solutions, they typically struggle with the complexity of modern biomedical data analysis. The comparative analysis highlights AI’s transformative capacity in precision diagnostics while clearly identifying key areas for advancement—particularly in enhancing model generalizability, establishing robust regulatory standards, and addressing ethical implications for real-world clinical integration.

Traditional drug delivery routes, such as oral and transdermal administration, also stand to benefit from AI. Oral drug delivery is the most common route of administration but often suffers from challenges like the poor solubility and low bioavailability of drugs. Transdermal drug delivery, which involves delivering drugs through the skin, is limited by the skin’s natural barrier function. AI can address these challenges by predicting the physicochemical properties that influence the drug absorption through these routes and optimizing drug formulations accordingly [55]. For example, machine learning algorithms can predict how different excipients and formulation techniques affect the solubility and dissolution rates of orally administered drugs. Similarly, AI can model how different drug formulations interact with the skin barrier, guiding the development of transdermal patches that improve drug penetration and absorption [56].

**Figure 2:**
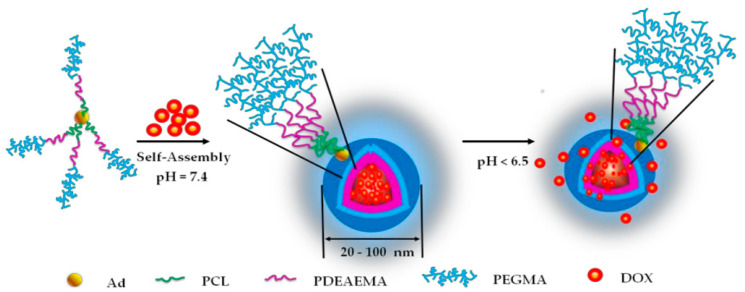
Illustration of pH-sensitive drug delivery micelles formed by adamantane-terminated [poly(ε-caprolactone)-block-poly(2-(diethylamino)ethyl methacrylate)-block-poly(poly(ethylene glycol) methyl ether methacrylate)]_4_ (Ad-(PCL-b-PDEAEMA-b-PPEGMA)_4_) triblock copolymers, demonstrating their Doxorubicin (DOX) encapsulation and controlled release mechanism in response to acidic environments. Reprinted with permission from Ref. [5]. cc by 4.0, https://doi.org/10.3390/polym10040443.

Controlled-release systems are designed to release drugs over an extended period, reducing the frequency of dosing and improving patient compliance. Targeted drug delivery systems aim to deliver drugs directly to the site of the disease, minimizing the exposure to healthy tissues. AI plays a crucial role in optimizing both controlled and targeted drug delivery by modeling how different materials and drug formulations influence the release kinetics and targeting specificity [57]. For instance, AI can integrate data from in vitro drug release studies, imaging data, and clinical outcomes to optimize the formulation of hydrogels, polymers, and nanoparticles for controlled and targeted drug release. In a study, AI models were used to predict the release profiles of drugs encapsulated in hydrogels based on the material’s properties and environmental conditions. This approach enabled the design of hydrogels that released drugs at a controlled rate in response to specific physiological triggers, such as changes in pH or temperature [58]. Figure 2 demonstrates the process of the drug encapsulation and release within micelles formed by the polymeric structure Ad-(PCL-b-PDEAEMA-b-PPEGMA)4. These micelles serve as nanocarriers for Doxorubicin (DOX), a widely used chemotherapy drug. The figure highlights how the micelles are formed through the self-assembly of the block copolymers, driven by hydrophobic interactions. The system exhibits pH-responsive behavior, where the release of DOX is triggered in acidic environments, such as tumor tissues, due to the protonation of the PDEAEMA block. This pH-dependent release mechanism enhances the targeted delivery of DOX, minimizing side effects and improving the therapeutic efficacy in cancer treatments [59].

### 2.5. AI in Regenerative Medicine Applications

Regenerative medicine seeks to restore compromised tissues and organs by augmenting innate biological repair mechanisms or deploying bioengineered constructs and cellular interventions. The incorporation of multimodal artificial intelligence significantly enhances this field by enabling the data-driven optimization of tissue scaffolds, precision stem cell applications, and targeted genome editing approaches. Through the sophisticated analysis of integrated imaging, molecular profiling, and proteomic datasets, AI systems facilitate the creation of tailored regenerative solutions with improved therapeutic outcomes [60].

Tissue engineering scaffolds serve as essential three-dimensional frameworks that facilitate cellular adhesion, growth, and specialization. Effective scaffold development requires a careful consideration of multiple parameters, such as structural integrity, pore network characteristics, and biological compatibility. Advanced multimodal AI systems enhance this process by synthesizing information from biomaterials research, histological patterns, and cellular response data to engineer scaffolds that precisely replicate the biomechanical and functional properties of natural tissues [61]. For example, AI models can analyze imaging data from tissues to determine their mechanical properties, such as elasticity and stiffness. This data can then be used to guide the selection of scaffold materials and architectures, ensuring that the scaffold provides the appropriate mechanical environment for cell growth. In bone tissue engineering, the AI-driven analysis of CT scans can help design scaffolds with the optimal porosity and mechanical strength for promoting bone regeneration [24]. Similarly, in skin tissue engineering, AI can analyze histological data to design scaffolds that support wound healing and tissue integration [62]. Table 4 provides a comparative analysis of AI-optimized biomaterials for tissue engineering applications, emphasizing their composition, functionality, and AI-driven enhancements [22,24,36,63,64]. Artificial intelligence has revolutionized biomaterial development by enabling the predictive modeling of material characteristics, the optimization of three-dimensional scaffold geometries, and the customization of formulations to enhance both biological integration and structural performance. Notably, advanced neural networks have successfully engineered hydrogel matrices with superior cellular binding properties and programmable degradation profiles, significantly improving tissue reconstruction outcomes. AI-driven generative models have also enabled the discovery of novel polymer composites with tunable porosity and mechanical properties suited for bone and cartilage regeneration. Furthermore, reinforcement learning algorithms have optimized 3D bioprinting parameters, ensuring a precise layer deposition and vascular network formation in engineered tissues. While AI-driven biomaterials demonstrate significant advancements in regenerative medicine, challenges remain in translating computational predictions into clinically viable solutions, necessitating further validation, regulatory standardization, and large-scale experimental studies.

Stem cell therapies are a cornerstone of regenerative medicine, offering the potential to replace damaged cells and tissues. However, the success of these therapies depends on a range of factors, including the selection of the appropriate stem cell type, the differentiation potential of the cells, and their ability to integrate with host tissues. Multimodal AI can optimize stem cell-based therapies by integrating genomic, proteomic, and clinical data to predict the behavior of stem cells in different biological environments [65]. Artificial intelligence enables precision stem cell selection by analyzing individual genetic profiles to identify optimal cell sources for patient-specific therapies. Machine learning algorithms can additionally forecast cellular differentiation pathways, predicting whether stem cells will develop into neural, cardiac, or bone-forming cells by analyzing microenvironmental signals and material characteristics. Research by Chen et al. demonstrated this capability through the AI-driven prediction of the mesenchymal stem cell osteogenic differentiation potential for skeletal tissue repair applications. By integrating genomic data with imaging data from bone tissues, the AI model could optimize the selection of stem cells and biomaterials for promoting bone formation [66].

**Figure 3:**
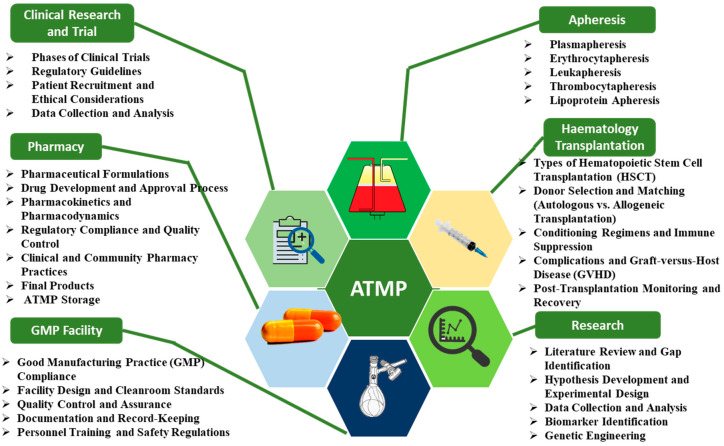
Visualizes the integrated workflow for advanced therapy medicinal products (ATMPs), highlighting their central position within a network of specialized clinical and research units. The schematic demonstrates how multidisciplinary hospital departments collectively contribute to ATMP development, manufacturing, and therapeutic implementation, underscoring the coordinated nature of these cutting-edge medical interventions.

CRISPR-based gene editing represents a transformative approach in regenerative medicine, enabling precise DNA modifications to address genetic disorders. The therapeutic efficacy of these interventions relies critically on both target specificity and the minimization of unintended genomic alterations. Multimodal artificial intelligence significantly improves editing precision by synthesizing multi-omics data to identify optimal target sequences and design efficient editing protocols [67]. Machine learning algorithms process extensive genomic information to pinpoint disease-relevant targets while predicting potential off-target activity through the computational modeling of CRISPR–cas9 interactions. A recent application demonstrated AI’s capability in muscular dystrophy therapy, where an integrated analysis of genomic and proteomic datasets enabled the identification of optimal editing sites while reducing off-target risks [68]. Figure 3 presents the complex organizational structure governing advanced therapy development, highlighting interdisciplinary coordination across healthcare sectors [69].

At the center of this framework is the advanced therapy medicinal products (ATMPs), surrounded by nodes that represent essential hospital units responsible for different aspects of ATMP development and application. This interconnected structure highlights the importance of interdisciplinary collaboration in successfully bringing ATMPs from conception to clinical use. Each unit, including research, regulatory affairs, and clinical applications, contributes to ensuring that these innovative therapies are effective, safe, and compliant with regulatory standards. Overall, the figure underscores the necessity of a cohesive approach to leverage the full potential of ATMPs in advancing patient care.

Multimodal AI is revolutionizing regenerative medicine through patient-specific therapeutic designs, synthesizing radiological, genomic, and clinical data to develop customized treatment solutions. This precision approach enhances therapeutic efficacy while mitigating potential adverse effects [70]. In articular cartilage repair, AI algorithms correlate MRI-derived structural data with genetic profiles to engineer biocompatible scaffolds optimized for individual tissue regeneration requirements. For myocardial repair, intelligent systems evaluate cardiac imaging and cellular biomarkers to select ideal stem cell-biomaterial combinations for targeted cardiac tissue restoration [71]. Such biologically tailored interventions mark a paradigm shift toward truly individualized medical care, where regenerative strategies are precisely adapted to each patient’s unique physiological characteristics and health profile.

### 2.6. Multimodal AI in Biomaterials Science

Multimodal AI, by integrating diverse biomedical data sources such as imaging, genomic sequences, and electronic health records, enables the precise engineering of biomaterials tailored to individual patient needs. This approach surpasses conventional computational techniques by uncovering complex, nonlinear relationships between biomaterial properties and biological responses. For instance, deep learning models trained on high-throughput experimental datasets can predict biomaterial–cell interactions, leading to the rational design of bioactive scaffolds that promote tissue regeneration. Similarly, AI-driven molecular simulations enhance drug delivery systems by optimizing nanoparticle formulations for controlled and targeted therapeutic release. Furthermore, multimodal AI facilitates the convergence of bioinformatics and material science, expediting the discovery of novel biomaterials with an enhanced biocompatibility and mechanical performance. This manuscript now provides a more in-depth discussion of these advancements, supported by case studies and comparative analyses of AI-based versus traditional approaches. By narrowing the scope to AI’s transformative impact on biomaterials science, our review offers a more focused and insightful contribution to the field, highlighting the practical implications, advantages, and challenges of integrating AI into biomaterials research and clinical translation.

## 3. AI-Powered Diagnostics and Precision Medicine

**Figure 4:**
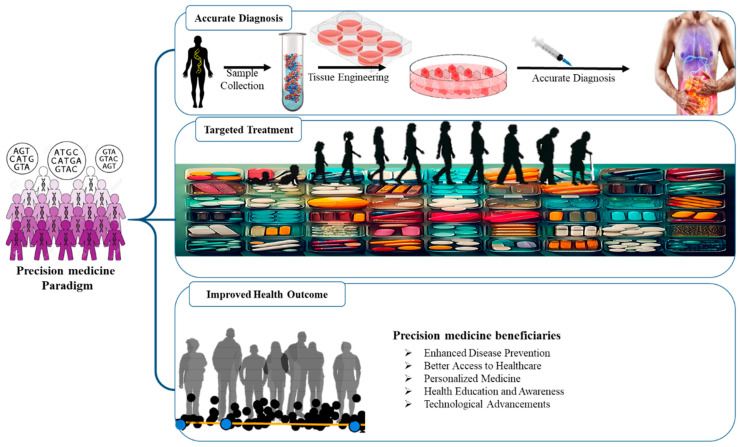
Illustrates the paradigm of precision medicine, showcasing contemporary strategies that involve evaluating different cancer treatment options, including chemotherapy, targeted therapies, and immunotherapies. These assessments typically utilize patient-derived cancer cells or models, such as spheroids and organoids, along with orthotopic murine xenograft models. The integration of AI-driven systems and technological platforms is expected to streamline and enhance this evaluation process, potentially leading to more rapid and personalized treatment strategies for cancer patients.

The integration of multimodal artificial intelligence has become a cornerstone of modern diagnostic and therapeutic innovation in healthcare. By synthesizing diverse biological datasets, including molecular profiles, medical imaging, and electronic health records, AI systems facilitate the creation of precise diagnostic methods and customized intervention strategies. This technological advancement represents a fundamental evolution in medical practice, where patient management is increasingly guided by the comprehensive analysis of individual pathophysiological characteristics. The following discussion examines AI’s transformative influence on both disease detection and tailored treatment development, highlighting its significant contributions to contemporary biomedical progress. Figure 4 depicts the precision medicine paradigm, emphasizing the evaluation of various cancer therapeutics, such as chemotherapy, targeted therapies, and immunotherapy [72]. By utilizing patient-derived tumor cells and advanced models like spheroids and organoids, this approach enables more accurate assessments of drug efficacy tailored to individual patient profiles. Additionally, the inclusion of orthotopic murine xenograft models further supports the translational potential of these therapies. The anticipated integration of AI-driven systems promises to accelerate this process by streamlining the data analysis and enhancing predictive accuracy, ultimately facilitating the development of personalized treatment regimens that improve patient outcomes in oncology.

### 3.1. Role of AI in Disease Diagnosis

Artificial intelligence is transforming clinical diagnostics by enabling a more precise identification and evaluation of complex pathologies, including malignancies, heart disease, and neurodegenerative conditions. While conventional diagnostic approaches remain valuable, their dependence on subjective assessments can result in inconsistent interpretations and therapeutic delays. Multimodal AI overcomes these challenges by synthesizing radiological, molecular, and patient history data to generate objective, evidence-based diagnostic conclusions [73].

Artificial intelligence has revolutionized medical image interpretation, with deep learning architectures demonstrating remarkable proficiency in analyzing radiological scans. Convolutional neural networks excel at processing MRI, CT, and radiographic images, detecting subtle pathological features that often elude human observation. This capability enables an earlier and more precise disease identification [74]. A prominent example is mammographic analysis, where CNNs trained on extensive labeled datasets can discern malignant patterns with an accuracy rivaling or surpassing expert radiologists [75]. Such systems provide real-time interpretation, dramatically decreasing diagnostic turnaround times. Clinical validation studies involving over 76,000 mammograms demonstrated AI’s superiority in reducing both false-positive and false-negative rates compared to conventional screening methods [76]. Comparable advancements have emerged in pulmonary oncology, with AI algorithms achieving an exceptional sensitivity in identifying malignant nodules on thoracic CT imaging [77].

Histopathology, the microscopic examination of tissue samples, is another area where AI is proving invaluable. Traditionally, pathologists analyze tissue biopsies manually, which can be time-consuming and prone to variability in interpretation. AI-powered digital pathology systems can analyze histological slides with greater consistency and speed, identifying abnormalities such as cancerous cells or inflammatory processes [78]. In prostate cancer diagnosis, for example, AI models have been trained to recognize patterns of cancerous growth in tissue samples with a high accuracy. These models can assist pathologists by providing second opinions or pre-screening slides for potential malignancies, thus reducing the workload on clinicians and improving diagnostic accuracy [79]. Similarly, in gastrointestinal pathology, AI has been employed to identify dysplastic lesions in colon biopsies, aiding in the early detection of colorectal cancer [80].

Artificial intelligence serves as a powerful bridge between molecular profiling and clinical decision-making, transforming complex genomic data into actionable diagnostic insights. As next-generation sequencing becomes more cost-effective, AI systems address the analytical challenges posed by massive genomic datasets by detecting disease-relevant mutations, transcriptional patterns, and molecular biomarkers. In oncology, machine learning algorithms distinguish pathogenic driver mutations from benign variants, while integrating these findings with radiological and clinical data for a comprehensive tumor characterization [81]. Commercial platforms exemplify this approach by cross-referencing individual genomic alterations with therapeutic databases to recommend evidence-based treatment regimens. Such systems enable truly precision oncology, where anticancer therapies are selected based on each tumor’s unique molecular fingerprint [82]. Artificial intelligence is increasingly valuable for detecting neurodegenerative conditions like Alzheimer’s and Parkinson’s diseases, where early identification remains clinically challenging due to an insidious symptom progression. Machine learning models demonstrate a particular efficacy in analyzing structural and functional neuroimaging Magnetic Resonance Imaging/Positron Emission Tomography (MRI/PET) alongside cognitive assessments to uncover preclinical indicators of neurological decline [83]. In Alzheimer’s disease, AI models have been trained to recognize patterns of brain atrophy and amyloid deposition in MRI and PET scans, providing earlier and more accurate diagnoses than conventional methods. In another study, an AI system was developed to analyze PET scans for early signs of Alzheimer’s disease, achieving an accuracy rate of over 90% in detecting the disease several years before clinical symptoms appeared [84]. Such advances in AI-driven diagnostics hold the promise of earlier interventions and improved outcomes for patients with neurodegenerative diseases.

**Figure 5:**
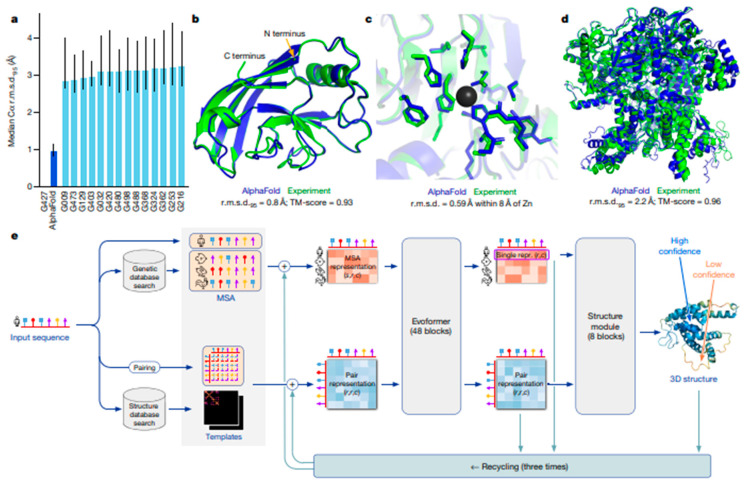
Demonstrates AlphaFold’s breakthrough protein structure prediction capabilities: (**a**) Benchmarking against CASP14 competitors (n = 87 domains) shows superior accuracy, with box plots representing median values and 95% confidence intervals across 10,000 bootstrap samples. (**b**) Structural alignment of prediction (blue) versus experimental determination (green) for target T1049 (6Y4F), excluding disordered C-terminal residues. (**c**) Precise modeling of zinc-coordination geometry in target T1056 (6YJ1), including correct side-chain placement despite omitting metal ions. (**d**) Accurate domain organization prediction for massive 2180-residue target T1044 (6VR4) achieved fully autonomously. (**e**) Schematic of AlphaFold’s neural network architecture, detailing dimensional parameters (Nseq = s, Nres = r, feature channels = c) and component interactions. Adapted from [85] under CC BY 4.0.

The role of protein structure predictions, particularly through advancements such as AlphaFold Ref. [85], is pivotal in the field of biomedicine and biomaterials development, as highlighted in this review. AlphaFold, an AI system developed by DeepMind, has demonstrated an unprecedented ability to predict the three-dimensional structures of proteins with remarkable accuracy, which is critical for understanding protein functions, interactions, and stability. In the context of biomaterials, accurate protein structure predictions enable researchers to design materials that closely mimic biological tissues, enhancing their compatibility and performance in tissue engineering and regenerative medicine. Furthermore, AlphaFold’s predictions support drug discovery by revealing potential binding sites and interactions at the molecular level, which can accelerate the development of targeted therapies. This capability is particularly relevant for designing biomaterials that interact with specific proteins or enzymes in drug delivery systems. The integration of AlphaFold’s predictions with other multimodal AI tools enhances our ability to manipulate and design proteins and biomaterials at the molecular level, offering transformative potential in creating highly tailored biomedical solutions. Figure 5 highlights AlphaFold’s remarkable capability to predict accurate protein structures, outperforming other top entries in the CASP14 competition [85]. The figure compares AlphaFold’s performance with other models using the median and confidence intervals, showing its superior predictive power. One example is the accurate alignment of AlphaFold’s predicted structure with an experimentally determined protein structure, demonstrating the model’s reliability in reproducing complex protein folds. Additionally, AlphaFold exhibits precise side-chain predictions in a zinc-binding site, despite not explicitly predicting the metal ion. In more complex cases, such as a 2180-residue protein, AlphaFold correctly predicts the domain organization, underscoring its ability to handle large and intricate protein structures. The figure also illustrates AlphaFold’s model architecture, showcasing how information flows between sequences, residues, and channels, which contributes to its high accuracy. These examples emphasize AlphaFold’s impact on structural biology, enabling more reliable protein modeling for research applications.

### 3.2. AI-Driven Precision Medicine

The transition to personalized healthcare marks a fundamental evolution in medical practice, replacing generalized treatment protocols with tailored therapeutic strategies based on individual patient profiles. Artificial intelligence serves as a critical enabler of this transformation by synthesizing multidimensional biological data—including genomic variations, protein expression patterns, and clinical histories—to guide therapeutic decision-making. Through the large-scale analysis of these complex datasets, machine learning algorithms can forecast patient-specific treatment responses, empowering clinicians to optimize therapeutic regimens for enhanced effectiveness and reduced complications [86]. Precision oncology has emerged as a prime beneficiary of AI-driven personalized medicine, addressing the critical challenge of tumor heterogeneity where histologically similar cancers demonstrate divergent treatment responses. Advanced machine learning algorithms synthesize multi-omics data from tumor biopsies with clinical and imaging datasets to generate patient-specific therapeutic recommendations [87]. These systems identify predictive biomarkers—including targetable mutations and immunogenic signatures—while forecasting individual drug sensitivity patterns. A recent breakthrough application involved gene expression-based machine learning models that accurately stratified breast cancer patients by chemotherapy response, enabling risk-adapted treatment protocols [88]. AI has also been used to optimize radiation therapy for cancer patients. By integrating imaging data with genomic information, AI can predict how tumors will respond to radiation, allowing for the design of radiation plans that maximize tumor destruction while minimizing the damage to surrounding healthy tissues [89]. AI-enhanced precision oncology significantly improves therapeutic efficacy while minimizing adverse effects through pharmacogenomic optimization. By analyzing genetic polymorphisms in drug metabolism pathways (e.g., CYP450 enzymes) and therapeutic targets, machine learning algorithms predict individual pharmacokinetic profiles, enabling customized dosing strategies [90]. This approach proves particularly impactful in cardiovascular care, where AI models incorporating CYP2C9/VKORC1 variants optimize warfarin dosing, reducing hemorrhage risks while maintaining anticoagulation efficacy [91]. In cancer therapeutics, these systems demonstrate a similar value by correlating tumor genomic landscapes with drug sensitivity patterns—as evidenced by recent non-small cell lung cancer research where an AI-guided therapy selection improved outcomes through mutation-specific treatment matching [92]. Cardiovascular medicine is leveraging AI to develop patient-specific management strategies for heart disease. Advanced algorithms process multimodal cardiac data, including electrophysiological signals, imaging biomarkers, and clinical histories, to stratify the individual risk for acute coronary events or cerebrovascular incidents. These predictive insights inform tailored therapeutic approaches, optimizing medication selection, lifestyle modifications, and interventional timing [93]. Notably, machine learning applications in arrhythmia prediction demonstrate particular clinical value; by detecting subtle ECG pattern abnormalities, these systems enable prophylactic measures ranging from pharmacological management to device implantation, substantially mitigating the probability of catastrophic cardiac events while enhancing prognostic outcomes [94].

Artificial intelligence is revolutionizing mental healthcare by enabling tailored interventions for heterogeneous psychiatric conditions, including depressive disorders, anxiety syndromes, and psychotic illnesses. Machine learning systems synthesize psychometric evaluations, neuroimaging findings, and genomic markers to forecast individual treatment responses, addressing the well-documented variability in therapeutic outcomes [95]. Particularly in major depression management, predictive algorithms analyze phenotypic and genetic factors to optimize the antidepressant selection, substantially reducing the conventional trial-and-error approach and accelerating symptom remission [96]. Despite this potential, implementation barriers persist, particularly regarding data protection requirements for sensitive health information and the need for explainable AI frameworks in clinical decision-making [97]. The continued progress in computational methods and multimodal data fusion promises to enhance these systems’ reliability and adoption, potentially transforming mental healthcare through precise diagnostic categorization and individualized treatment protocols [98].

### 3.3. Advanced Predictive Modeling in Healthcare Through Machine Learning

Machine learning has emerged as a transformative force in healthcare predictive analytics, enabling the data-driven forecasting of clinical trajectories. These computational approaches leverage historical patient data to generate probabilistic assessments of disease progression, therapeutic efficacy, and adverse event risks—facilitating proactive medical interventions. When combined with multimodal AI architectures, predictive models gain an enhanced capability to process heterogeneous datasets spanning molecular profiles, medical imaging, and electronic health records. This integration enables the development of sophisticated prognostic tools that deliver a personalized risk stratification with improved accuracy, ultimately supporting precision medicine initiatives.

Various machine learning algorithms, such as logistic regression, decision trees, Support Vector Machines (SVMs), and deep learning models, have been employed in predictive healthcare. Logistic regression, a fundamental method in medical statistics, remains popular for its simplicity and interpretability in predicting binary outcomes, such as disease presence or absence [99]. However, with the increasing complexity of available healthcare data, more sophisticated models like decision trees and SVMs, which can handle nonlinear relationships, are being adopted. Deep learning models, particularly convolutional neural networks (CNNs) and recurrent neural networks (RNNs), are proving to be even more effective at making predictions when complex, high-dimensional datasets are involved. CNNs are particularly useful for analyzing medical imaging data, while RNNs are often employed for time-series data, such as monitoring patient vitals [100]. These models can learn hierarchical patterns in data, automatically identifying significant features that would be difficult for clinicians to notice manually. An example of machine learning in predictive healthcare is the prediction of patient readmission rates, a critical issue in hospital management. Predicting which patients are at risk of readmission allows healthcare providers to intervene proactively. In one study, a machine learning algorithm was able to predict 30-day hospital readmissions for heart failure patients by analyzing factors like previous admissions, medications, and vital signs, achieving a predictive accuracy significantly higher than traditional statistical methods [101]. Another example involves predicting the progression of chronic diseases, such as diabetes or kidney disease, by analyzing clinical data and lab results. ML models can identify early signs of disease progression, allowing for timely interventions to prevent complications [102].

Predicting the onset of diseases at their earliest stages is a key area where ML models have demonstrated considerable success. One of the most significant applications is in the early prediction of cancer. Using ML algorithms trained on large datasets that include clinical records, genomic data, and medical images, AI can predict the likelihood of cancer development before clinical symptoms appear. For instance, an AI system developed to analyze mammograms for breast cancer risk was able to predict the onset of cancer up to five years in advance with an accuracy higher than that of human radiologists [103]. A similar predictive model was applied to Alzheimer’s disease, where machine learning algorithms analyzed brain scans and genetic markers to predict the onset of the disease before clinical symptoms became evident. A study conducted by a scientist group demonstrated that a deep learning model could predict Alzheimer’s disease from PET scans up to six years before an official diagnosis, with an accuracy of over 90% [104]. Early prediction enables patients and healthcare providers to take preventive measures, potentially delaying the progression of the disease.

Despite its transformative potential, predictive analytics in healthcare faces several critical challenges that must be addressed. A fundamental limitation stems from the training data quality, as many models rely on retrospective datasets with a limited generalizability across diverse populations. When certain demographic groups are underrepresented, algorithmic biases can emerge, potentially exacerbating healthcare disparities for marginalized communities [105]. Model interpretability presents another hurdle, especially for complex deep learning systems whose decision-making processes remain opaque. While emerging techniques like SHAP and LIME offer post hoc explanations for model outputs [106], further advances are needed to meet clinical transparency requirements. Ethical considerations around data privacy and informed consent also demand careful attention, particularly regarding their compliance with international standards like GDPR [107]. Looking ahead, incorporating multimodal data streams from genomic profiles to wearable device metrics while employing privacy-preserving approaches such as federated learning [108] will be crucial for developing robust, equitable predictive systems that account for the full spectrum of health determinants.

### 3.4. Multimodal Data Fusion for Enhanced Diagnostic Accuracy

Multimodal AI, which integrates diverse data sources such as medical images, genomic data, and clinical records, is revolutionizing diagnostics by providing a more comprehensive and nuanced understanding of patient health. Data fusion refers to the process of combining information from different modalities to enhance the accuracy and reliability of diagnostic predictions. In biomedicine, leveraging multimodal data fusion allows AI systems to provide richer, more precise diagnostic insights than any single modality could offer on its own.

Single-modality diagnostics, where only one type of data is analyzed (e.g., medical imaging or genetic data), often suffer from limitations that can reduce diagnostic accuracy. For example, relying solely on medical images may miss underlying molecular or genetic abnormalities that contribute to disease progression. Similarly, using only genomic data may overlook anatomical or functional changes visible in imaging studies. Each modality provides only a partial view of the patient’s condition, leading to potential misdiagnoses or incomplete assessments [109]. An illustrative example is the diagnosis of brain tumors. While imaging techniques such as MRI are excellent at detecting the physical presence and size of a tumor, they cannot provide information on the tumor’s genetic mutations or molecular characteristics, which are crucial for determining the most effective treatment. Integrating imaging data with genomic information can significantly enhance diagnostic accuracy by providing both structural and molecular insights into the tumor [110].

Multimodal data integration combines complementary data types to enhance diagnostic precision through comprehensive analytical models. This synergistic approach in AI-based diagnostics merges radiological, molecular, and clinical datasets to minimize diagnostic errors while improving detection accuracy across various pathologies [111]. In oncological applications, the convergence of radiomic features from advanced imaging with molecular profiling data enables a more precise tumor characterization, prognostic stratification, and therapeutic personalization. The research demonstrates a superior predictive capability when combining CT-derived radiomics with transcriptomic data for forecasting treatment responses in pulmonary malignancies, outperforming single-modality analytical approaches [112].

The integration of imaging and genomic data represents a transformative frontier in precision diagnostics. By correlating radiological patterns with molecular profiles, AI systems can predict disease susceptibility, genetic mutations, and therapeutic responses with unprecedented accuracy. In breast oncology, the combined analysis of mammographic features and genomic signatures enables a reliable prediction of tumor recurrence risk, guiding critical treatment decisions regarding the chemotherapy necessity [113]. Similarly, for neurodegenerative conditions, AI models synthesizing PET imaging with APOE4 genotyping demonstrate a remarkable capability in preclinical Alzheimer’s detection, facilitating early intervention opportunities [114].

Multimodal AI applications now span diverse medical specialties, demonstrating superior performances to conventional diagnostic approaches. Cardiovascular risk assessment exemplifies this advancement, where an integrated analysis of echocardiograms, electronic health records, and genetic data achieves more accurate heart failure predictions than established risk-scoring systems [115]. A landmark study in preventive cardiology showed such multimodal systems surpassing traditional metrics like the Framingham Risk Score in forecasting acute cardiovascular events [116]. Future development focuses on incorporating emerging data streams—from wearable biosensors to environmental factors—while overcoming challenges of healthcare data interoperability and maintaining rigorous ethical standards for data privacy and algorithmic transparency [117,118].

## 4. Wearable Technologies and Real-Time Health Monitoring

### 4.1. AI Integration with Wearable Devices

**Figure 6:**
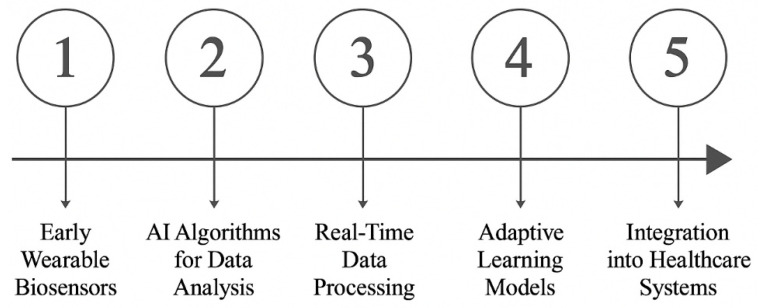
Chronological progression of AI-enhanced wearable biosensor networks (WAIBNs).

Modern wearable health technologies have evolved into intelligent monitoring systems through artificial intelligence integration, transitioning from basic biometric tracking to sophisticated health analysis platforms. These AI-enhanced devices process continuous physiological data streams to detect emerging health patterns, forecast potential risks, and deliver customized health recommendations, establishing a new paradigm of proactive, data-driven wellness management. Figure 6 outlines the significant milestones in the evolution of AI-assisted wearable biosensor networks (WAIBNs), highlighting the technological advancements that have facilitated their integration into healthcare [119]. The timeline showcases the transition from basic biosensors to sophisticated, AI-enhanced systems capable of real-time data analysis and interpretation. These technological advancements facilitate the uninterrupted tracking of vital health metrics, significantly improving clinical monitoring and therapeutic management. A sophisticated machine learning integration transforms raw biometric data into actionable health intelligence, enabling preemptive medical interventions tailored to individual needs. Collectively, these innovations demonstrate AI’s transformative impact on wearable health technologies, driving progress in telemedicine and precision healthcare delivery.

Contemporary wearable technologies span consumer-grade fitness trackers to clinical-grade biosensors, collectively enabling the continuous tracking of vital physiological parameters. These devices capture critical health metrics, including cardiovascular function, metabolic indicators, and behavioral patterns, generating longitudinal datasets essential for identifying health trends and enabling proactive interventions [120]. Research demonstrates their particular value in chronic disease management, where real-time physiological streaming enhances clinical decision-making [121]. The incorporation of artificial intelligence transforms these devices from passive data collectors to active diagnostic assistants through advanced signal processing and pattern recognition. Modern wearables employ sophisticated machine learning architectures to derive clinically meaningful insights from continuous biometric streams. Deep learning models excel at detecting subtle pathological patterns, such as cardiac arrhythmias in ECG waveforms or early hypertension risk factors from blood pressure trends [122,123]. These systems enable truly personalized healthcare by analyzing individual health patterns to generate tailored lifestyle recommendations and medication adherence prompts [124]. In diabetes care, AI-enhanced continuous glucose monitors provide real-time glycemic feedback while analyzing behavioral data to optimize dietary and activity suggestions [125], representing a paradigm shift in chronic disease self-management. Despite their potential, AI-powered wearables face significant adoption barriers including data security vulnerabilities and a lack of standardization across device ecosystems [126,127]. Addressing these limitations requires robust encryption protocols and unified data frameworks compliant with healthcare regulations. Future advancements will focus on a multidimensional health analysis incorporating psychosocial and environmental determinants through next-generation sensors and adaptive machine learning models, promising to redefine preventive healthcare delivery through increasingly sophisticated wearable health ecosystems.

Wearable devices encompass a range of technologies, including smartwatches, fitness trackers, and medical-grade sensors. These devices can monitor various physiological parameters, such as heart rate, blood pressure, blood glucose levels, sleep patterns, and physical activity. The continuous monitoring capabilities of wearables facilitate the collection of longitudinal health data, which is critical for understanding health trends and making timely interventions [120]. For instance, a study highlighted that wearable devices could effectively monitor patients with chronic diseases, providing real-time data that helps healthcare providers make informed decisions [121]. The integration of AI into these wearables enhances their functionality by enabling a data analysis and interpretation that go beyond simple metrics.

The integration of AI algorithms allows wearables to not only collect but also analyze data in real-time. Machine learning techniques can identify patterns and anomalies in the data, offering insights that can inform healthcare decisions. For example, wearable ECG monitors use AI algorithms to detect arrhythmias, providing early warnings to both patients and healthcare providers [122]. These systems have shown high accuracy rates in identifying abnormal heart rhythms, which is crucial for preventing serious cardiovascular events. Deep learning models are particularly effective in processing the complex data generated by wearable devices. These models can learn from large datasets to improve their predictive capabilities. For instance, researchers developed a deep learning model that could predict the risk of hypertension based on data collected from wearable blood pressure monitors. The model analyzed historical data, lifestyle factors, and real-time measurements to identify individuals at high risk of developing hypertension [123].

The AI integration with wearable devices allows for personalized health management by tailoring interventions based on a real-time data analysis. For example, wearables can provide personalized feedback and recommendations for physical activity, diet, and medication adherence based on the individual’s health data. This personalization enhances patient engagement and promotes healthier lifestyles [124]. In diabetes management, wearable devices equipped with continuous glucose monitors (CGMs) and AI algorithms can provide real-time feedback on blood glucose levels. These systems can alert patients when their levels are too high or too low, enabling timely interventions. Moreover, AI can analyze dietary patterns and physical activity levels to suggest lifestyle changes that can help maintain optimal blood glucose levels [125]. Despite the promising benefits of AI integration with wearable devices, several challenges must be addressed. Data privacy is a significant concern, as continuous monitoring involves collecting sensitive health information. Ensuring the security of this data is paramount to maintaining patient trust and complying with regulations such as the Health Insurance Portability and Accountability Act (HIPAA) [126]. Another challenge is the standardization of data collected from various wearable devices. Inconsistent data formats and metrics can hinder interoperability and the ability to aggregate data from multiple sources. Standardization efforts are essential to create a unified ecosystem that enhances the effectiveness of AI algorithms [127]. Looking forward, the future of AI-integrated wearables lies in developing more sophisticated algorithms that can analyze not only physiological data but also contextual factors, such as emotional well-being and social determinants of health. The incorporation of advanced sensors and machine learning models will enable wearables to provide even deeper insights into health, ultimately transforming the way healthcare is delivered.

### 4.2. AI-Enhanced Remote Patient Monitoring

The adoption of remote patient monitoring (RPM) systems has accelerated significantly, driven by technological advancements and pandemic-induced healthcare transformations. These platforms utilize interconnected wearable sensors, mobile health applications, and virtual care interfaces to enable continuous physiological tracking beyond clinical environments. AI integration augments RPM capabilities through advanced data analytics, facilitating early anomaly detection and personalized care recommendations while maintaining the care quality across distances. Modern RPM architectures typically incorporate three key elements: biometric wearables for vital sign acquisition (e.g., cardiac rhythm and oxygen saturation), patient-facing applications for data transmission, and clinician portals for centralized monitoring [128]. Such systems prove particularly valuable for chronic disease management, exemplified by heart failure protocols that track dynamic parameters like daily weight fluctuations and cardiopulmonary metrics, enabling preemptive clinical interventions before acute decompensation occurs [129].

The incorporation of AI into RPM systems significantly enhances their functionality. AI algorithms can analyze data collected from wearables to identify trends and anomalies that may indicate potential health issues. For example, an AI system can monitor heart failure patients’ weight and fluid intake data to predict exacerbations, allowing healthcare providers to intervene before hospitalization is necessary [130]. A study demonstrated that AI-powered RPM systems reduced hospital readmission rates for heart failure patients by over 30%, showcasing the effectiveness of these technologies [131]. Intelligent risk stratification represents a key advantage of AI-enhanced monitoring systems, which synthesize multi-source data streams from wearables, electronic records, and patient-generated health metrics. This analytical capability allows clinicians to precisely identify vulnerable patient subgroups needing prioritized care, thereby optimizing resource allocation and enhancing healthcare system efficiency [132]. Despite these advantages, significant implementation barriers must be overcome to achieve broad clinical adoption. One significant challenge is patient engagement and adherence to using wearable devices and monitoring systems. Studies have shown that patient engagement decreases over time, which can limit the effectiveness of RPM systems [133]. Strategies to improve patient adherence include user-friendly interfaces, educational resources, and regular communication with healthcare providers. Data privacy and security concerns also pose challenges. RPM systems collect sensitive health information, making it essential to implement robust security measures to protect patient data. Ensuring compliance with regulations, such as the HIPAA, is crucial to maintaining patient trust and confidentiality [134]. Another challenge is the integration of RPM systems into existing healthcare workflows. Healthcare providers may face difficulties in adapting to new technologies and incorporating RPM data into clinical decision-making. Training and support for healthcare professionals are essential to facilitate the successful implementation of RPM systems [135]. The evolution of remote patient monitoring systems shows tremendous potential as AI algorithms and wearable technologies advance. Next-generation platforms will deliver increasingly precise predictive analytics, enabling more accurate health assessments and personalized interventions [136]. The convergence of RPM with telehealth solutions will create dynamic care ecosystems, allowing immediate clinician–patient interactions when abnormalities are detected. The expansion into diverse care settings from home-based care to rehabilitation centers promises to democratize the access to continuous monitoring while reducing healthcare expenditures [137]. This synergistic integration of AI and connected health technologies is ushering in a paradigm shift toward anticipatory, precision medicine—one that prioritizes prevention, personalization, and health system sustainability through data-driven care delivery models.

### 4.3. Use of Multimodal Data for Continuous Health Surveillance

Modern biosensor devices have ushered in a new era of continuous physiological tracking through sophisticated multimodal data integration. These systems capture comprehensive health metrics, including cardiopulmonary function, metabolic indicators, and movement patterns, while simultaneously recording environmental exposures and behavioral patterns [138]. This multidimensional approach enables unprecedented insights into individual health trajectories, allowing for preemptive clinical interventions before acute manifestations occur.

Multimodal data refers to information collected from various sources, providing a holistic view of an individual’s health. In the context of wearable technologies, this data can include the following:

**Physiological Data:** Collected from devices such as smartwatches, fitness trackers, and medical sensors that monitor vital signs.

**Environmental Data:** Information about the patient’s surroundings, such as the air quality, temperature, and humidity, which can impact health.

**Behavioral Data:** Insights into lifestyle factors, including physical activity levels, sleep patterns, and dietary habits.

**Social Data:** Contextual information regarding social interactions and support systems, which can influence mental and emotional well-being [138].

The true power of wearable technologies lies in their ability to correlate disparate health determinants. Continuous glucose monitoring systems exemplify this capability, combining biochemical measurements with activity logs and nutritional data to optimize diabetes management [139,140]. Research confirms such integrated approaches yield superior glycemic control compared to conventional monitoring methods [141]. Similarly, advanced cardiac monitors now analyze electrocardiographic signals in the context of sleep quality and exertion levels, providing nuanced cardiovascular assessments impossible through isolated measurements [142].

Machine learning transforms raw wearable data into clinically actionable intelligence through pattern recognition and predictive modeling. Deep learning architectures process complex physiological time-series to forecast adverse events like atrial fibrillation episodes or COPD exacerbations [143,144]. These systems detect subtle preclinical signatures often imperceptible to human observers, enabling truly proactive medicine. The integration of convolutional neural networks allows for the simultaneous interpretation of multiple biosignals while maintaining temporal relationships critical for an accurate diagnosis [142].

Real-world implementations demonstrate wearable technology’s transformative potential. COPD management systems combining pulmonary function metrics with environmental and activity data have significantly reduced acute hospitalizations through early warning algorithms [144]. Cardiovascular studies utilizing multimodal wearables show improved arrhythmia detection rates and treatment outcomes compared to standard monitoring [145]. These successes validate the clinical value of continuous, integrated health surveillance systems in chronic disease management.

While the integration of multimodal data holds great promise for continuous health surveillance, several challenges must be addressed. One significant issue is the interoperability of devices and systems. The diverse range of wearables on the market often produces data in different formats, making it challenging to aggregate and analyze information effectively [146]. The standardization of data formats and communication protocols is essential to create a seamless ecosystem for health monitoring. Another challenge is ensuring the accuracy and reliability of the data collected by wearable devices. Variability in sensor performance and environmental factors can influence data quality. Therefore, implementing robust data validation and calibration processes is critical to ensure that the information used for health surveillance is accurate and actionable [147].

The future of continuous health surveillance through wearable technologies lies in enhancing data integration capabilities and leveraging advances in AI and machine learning. As wearable devices become more sophisticated, they will be able to collect an even wider array of data types, including biomarkers from sweat or saliva, genomic data, and mental health assessments through mobile applications [148]. Furthermore, the use of federated learning, a machine learning technique that allows models to be trained across decentralized data sources while preserving privacy, has the potential to enhance predictive analytics without compromising patient confidentiality [149]. This approach can facilitate the development of more accurate models that consider diverse patient populations and health conditions. Just above, Table 5 offers a comparison summarizing the key elements covered in this review, illustrating the advancements and challenges of multimodal AI in biomedicine and biomaterials, along with its implications for personalized healthcare. This table provides a comprehensive overview of the benefits, current limitations, and future potential of multimodal AI in transforming biomedicine and biomaterials science for personalized healthcare applications.

### 4.4. Impact of AI on Preventive Healthcare

Artificial intelligence is redefining preventive medicine through advanced risk prediction and early intervention capabilities. Machine learning algorithms analyze comprehensive health datasets to identify subtle patterns preceding disease onset, enabling the proactive management of chronic conditions [150]. In oncology, deep learning systems demonstrate superior accuracy in early cancer detection through a medical imaging analysis, significantly improving the diagnostic precision for breast and colorectal malignancies [151,152]. This predictive capacity shifts healthcare from reactive treatment to anticipatory care, simultaneously enhancing outcomes and reducing long-term costs.

Sophisticated AI models synthesize diverse health indicators, including genetic predispositions, lifestyle factors, and physiological metrics, to generate individualized risk assessments [152,153]. During recent public health crises, these systems proved invaluable for forecasting the infection spread by processing mobility patterns and healthcare utilization data [154]. The technology now enables personalized prevention strategies, from genomic-based screening recommendations to AI-powered health coaching applications that analyze wearable device data for real-time lifestyle interventions [155,156,157]. This granular approach to risk mitigation represents a fundamental advancement in population health management.

While promising, AI-enabled prevention faces significant hurdles in data quality and privacy protection. Incomplete or non-representative health datasets can introduce dangerous biases into predictive models, necessitating the rigorous standardization of training data [158]. Simultaneously, the sensitive nature of health information demands robust security measures and strict compliance with patient privacy regulations like the HIPAA [159]. Addressing these challenges requires a careful balance between data accessibility for AI development and the protection of individual rights.

The next frontier of AI prevention integrates emerging data streams from wearable biosensors, social determinants, and unstructured clinical narratives [158,160]. Natural language processing breakthroughs will unlock insights from physician notes and patient-reported outcomes, while federated learning approaches may resolve privacy concerns [161]. As these technologies mature, they promise to establish a new paradigm of continuous health optimization—shifting healthcare systems from episodic treatment to sustained wellness maintenance through intelligent, data-driven prevention strategies.

## 5. Ethical, Regulatory, and Scalability Challenges

### 5.1. Ethical Concerns in AI Applications

**Figure 7:**
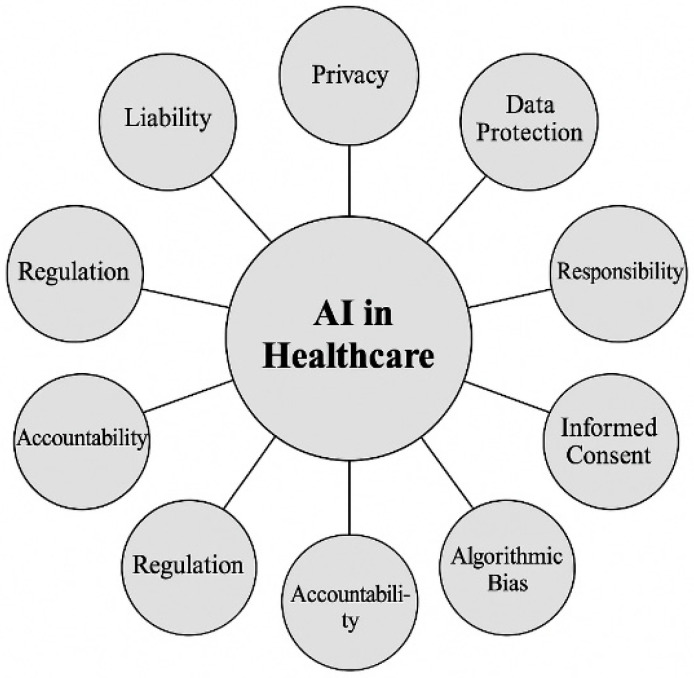
Ethical and legal dilemmas in AI for healthcare.

As artificial intelligence (AI) continues to permeate various sectors, including biomedicine, ethical concerns have emerged that warrant critical examination. The implementation of AI technologies in healthcare raises numerous ethical questions regarding patient autonomy, consent, bias, transparency, and accountability. Addressing these ethical concerns is essential to ensuring that AI applications enhance, rather than undermine, patient care and public trust. Figure 7 highlights the multifaceted ethical and legal challenges surrounding the implementation of artificial intelligence (AI) in healthcare [162]. Key issues include patient privacy and data security, as the use of AI often necessitates access to sensitive medical information. Additionally, there are concerns regarding algorithmic bias, which can lead to unequal treatment outcomes among diverse patient populations. The figure also underscores the need for transparent decision-making processes, as AI-driven recommendations can lack interpretability. Moreover, the regulatory landscape remains complex, necessitating a framework that addresses liability and accountability in cases of AI-related errors. Collectively, these dilemmas underscore the importance of integrating ethical considerations into the development and deployment of AI technologies in healthcare settings.

One of the most significant ethical issues in AI applications is the impact on patient autonomy. In traditional healthcare settings, patients are often involved in decision-making processes regarding their treatment options. However, with the increasing use of AI-driven diagnostic tools and treatment recommendations, there is a risk that patients may become passive recipients of care, relying on algorithms to make decisions for them [163]. This shift raises concerns about informed consent, as patients may not fully understand how AI systems work, the data being used, or the implications of AI-driven decisions. Informed consent is a foundational ethical principle in healthcare, requiring that patients are fully aware of the nature and consequences of their treatment options [164]. With the complexity of AI algorithms, ensuring that patients understand these systems is challenging. Healthcare providers must take proactive steps to educate patients about AI technologies, including how they are used in diagnosis and treatment, to ensure that patients can make informed choices regarding their care.

Bias in AI algorithms is another critical ethical concern. AI systems are trained on historical data, which may contain inherent biases that reflect societal inequalities. If these biases are not addressed, AI applications can perpetuate existing disparities in healthcare outcomes [165]. For instance, a study found that certain algorithms used in clinical settings exhibited racial bias, leading to the underdiagnosis and undertreatment of minority groups [166]. This is particularly concerning in biomedicine, where the equitable access to treatments and diagnostic tools is essential for improving patient outcomes. To combat bias, researchers and developers must prioritize diversity in training datasets, ensuring that AI algorithms are exposed to a wide range of demographic and clinical variables. Furthermore, implementing regular audits and evaluations of AI systems can help identify and mitigate biases, promoting fairness in healthcare delivery [167].

The “black-box” nature of many AI algorithms poses challenges to transparency and explainability. When AI systems generate recommendations or diagnoses, it can be difficult for healthcare providers and patients to understand the underlying reasoning behind these decisions. This lack of transparency can undermine trust in AI applications and raise ethical concerns regarding accountability [168]. To address these issues, it is crucial to develop explainable AI models that provide insights into their decision-making processes. Explainable AI allows healthcare professionals to interpret AI outputs and communicate them effectively to patients, fostering a collaborative decision-making environment [169]. Ensuring transparency not only enhances trust in AI systems but also empowers healthcare providers to critically evaluate AI recommendations, ultimately improving patient care.

The question of accountability and liability in AI applications is another ethical dilemma. When an AI system makes a mistake, such as providing an incorrect diagnosis or treatment recommendation, determining who is responsible can be complex. Is it the healthcare provider, the software developer, or the institution that deployed the AI system? This ambiguity can hinder efforts to address errors and learn from them [170]. Establishing clear guidelines for accountability in AI applications is essential. Regulatory bodies and healthcare institutions must work collaboratively to define standards for AI deployment, including protocols for error reporting and accountability mechanisms. Furthermore, incorporating human oversight in AI decision-making processes can help mitigate risks associated with erroneous outputs and clarify responsibility in case of adverse outcomes [171].

The ethical implications of data privacy and security are paramount when implementing AI in biomedicine. AI systems often rely on large datasets that contain sensitive patient information. Ensuring the confidentiality and security of this data is crucial to maintaining patient trust and complying with regulations such as the Health Insurance Portability and Accountability Act (HIPAA) [172]. Breaches in data security can have severe consequences for patients, including identity theft and the loss of confidentiality. To safeguard patient data, healthcare organizations must implement robust cybersecurity measures and establish strict protocols for data handling. Additionally, patients should be informed about how their data will be used and stored, empowering them to make informed choices about their participation in AI-driven healthcare initiatives [173]. The ethical concerns surrounding AI applications in biomedicine are multifaceted and complex. Addressing these issues requires a collaborative effort from healthcare providers, researchers, policymakers, and technology developers. By prioritizing patient autonomy, mitigating bias, ensuring transparency, clarifying accountability, and safeguarding data privacy, the healthcare sector can harness the potential of AI technologies while upholding ethical standards that prioritize patient welfare and trust.

### 5.2. Regulatory Frameworks for AI-Driven Systems

The integration of AI into biomedicine demands comprehensive regulatory oversight to ensure patient safety while fostering innovation. As these technologies advance, governing bodies must develop adaptive frameworks that address unique challenges in clinical validation, algorithmic transparency, and ethical implementation. Effective regulations will balance technological progress with rigorous standards for efficacy, safety, and equitable access across healthcare systems. Global regulatory approaches are emerging to govern medical AI applications, with distinct regional strategies taking shape. The U.S. FDA has implemented specialized pathways for AI-based medical devices through its Digital Health Innovation Action Plan, emphasizing rigorous premarket evaluations and ongoing performance monitoring [174]. Meanwhile, the EU’s proposed Artificial Intelligence Act introduces risk-based classifications for AI systems, with stringent requirements for healthcare applications, including mandatory clinical validation, auditability, and maintained human oversight [175]. These evolving frameworks reflect the international community’s commitment to responsible AI adoption in medicine.

A risk-based regulatory approach is essential for managing AI technologies in healthcare. By classifying AI applications according to their potential risks, regulatory bodies can tailor oversight measures accordingly. For example, high-risk applications, such as AI algorithms used for diagnostic purposes, may require rigorous pre-market testing and ongoing monitoring to ensure safety and effectiveness [176]. Conversely, lower-risk applications, such as AI-driven wellness apps, may be subject to less stringent regulations. This approach enables regulatory bodies to allocate resources effectively while fostering innovation in lower-risk areas. A risk-based framework also encourages transparency in AI algorithms, enabling healthcare providers to assess their reliability and suitability for clinical use [177]. The effective governance of medical AI requires multi-stakeholder collaboration to develop balanced regulatory approaches. Involving clinicians, technologists, patients, and policymakers ensures frameworks address real-world needs while maintaining ethical standards [178]. Regulatory agencies like the FDA and EMA are pioneering inclusive consultation models that incorporate diverse perspectives into policy development, fostering transparency in AI validation and deployment processes [179]. Such participatory approaches help align technological capabilities with clinical priorities while building public confidence. Regulatory systems must maintain adaptability to keep pace with AI’s rapid evolution in healthcare. Continuous framework refinement is essential to address emerging challenges in algorithm validation, data governance, and clinical integration [180]. Robust postmarket surveillance mechanisms enable an ongoing evaluation of AI system performance, identifying potential safety issues or performance drifts in operational environments [181]. This dynamic regulatory posture ensures patient protections evolve alongside technological advancements. International regulatory harmonization presents both a challenge and opportunity for global health innovation. Divergent national standards create compliance complexities for AI developers while potentially delaying patient access to beneficial technologies [182]. Initiatives like the WHO’s digital health programs promote cross-border collaboration, working toward a consensus on core principles for medical AI evaluation and oversight [183]. Establishing universal standards for high-risk applications while allowing regional adaptations could accelerate the responsible global adoption of transformative healthcare AI solutions.

### 5.3. Challenges in Scaling AI Systems for Clinical Use

The widespread deployment of AI systems in healthcare faces significant technical hurdles, primarily stemming from fragmented data ecosystems. Disparate electronic health record systems with incompatible data structures create substantial obstacles for algorithm training and validation [184]. Standardization initiatives like FHIR aim to establish unified data exchange protocols, while common data models help harmonize information across platforms [185,186]. Equally critical is ensuring algorithmic robustness through rigorous testing on diverse, representative datasets to mitigate biases and enhance generalizability across patient populations [187,188,189]. A successful AI adoption requires a careful alignment with clinical operations to minimize workflow disruptions. Resistance from healthcare professionals often stems from poorly designed interfaces and inadequate training programs [190]. Developing intuitive systems with seamless EHR integration, coupled with comprehensive clinician education initiatives, can facilitate smoother transitions [191,192]. Financial constraints present another major barrier, particularly for resource-limited settings. Strategic partnerships between healthcare providers, academic centers, and technology developers can create cost-sharing models that make AI implementation more accessible [193,194,195].

Professional skepticism and patient concerns represent significant adoption barriers that demand proactive management. Clinicians require transparent demonstrations of AI’s assistive (rather than replacement) role in clinical decision-making [196]. Including healthcare providers in development cycles and showcasing validated use cases helps build confidence in these technologies [197,198]. Patient education initiatives must clearly communicate AI’s complementary role in care delivery while addressing privacy concerns and algorithmic transparency [199,200,201]. Realizing AI’s full potential in clinical settings demands coordinated strategies addressing technical, financial, and human factors. Prioritizing interoperable data infrastructure, clinician-centered design, and ongoing performance monitoring creates a foundation for successful scaling. Simultaneously, innovative funding models and stakeholder engagement initiatives can overcome resource limitations and cultural resistance. By adopting this multifaceted approach, healthcare systems can responsibly integrate AI to enhance diagnostic accuracy, therapeutic development, and patient outcomes while maintaining the human touch in medicine.

### 5.4. Addressing Data Privacy and Security

The implementation of multimodal AI in biomedicine raises critical data protection challenges given its reliance on sensitive health information. Maintaining data integrity while preserving patient confidentiality requires sophisticated security frameworks, particularly as healthcare systems become increasingly digitized. Breaches risk severe consequences, including medical identity theft and the erosion of public trust in medical AI systems [202]. Regulatory frameworks like the HIPAA and GDPR establish essential guidelines for data handling, mandating strict consent protocols, access controls, and security measures that organizations must rigorously implement [203].

The development of effective AI models necessitates cross-institutional data sharing, creating tension between research needs and privacy protection. Privacy-preserving computational methods offer viable solutions to this challenge. Differential privacy techniques enable an aggregate analysis while obscuring individual identities through strategic data perturbation [204,205]. Federated learning architectures allow collaborative model training across institutions without direct data exchange, maintaining information security while advancing AI capabilities [206]. These approaches demonstrate how technological innovation can balance research progress with ethical obligations.

Healthcare organizations face escalating threats from sophisticated cyberattacks targeting valuable medical data. Comprehensive defense strategies must incorporate end-to-end encryption, continuous vulnerability assessments, and staff cybersecurity training [207,208]. Proactive measures like real-time network monitoring and automated threat detection systems can identify breaches early, while detailed incident response plans ensure rapid containment [209]. Such multilayered security approaches are becoming essential infrastructure for AI-enabled healthcare delivery.

Transparent data practices form the foundation of ethical AI implementation in medicine. Patients deserve clear communication about how their information will be used, protected, and potentially shared [210]. Organizations should prioritize informed consent processes and patient education initiatives to foster trust in AI applications [211,212]. Regulatory compliance tools can automate the monitoring of data handling practices, while ongoing collaboration with legal experts ensures the alignment with evolving privacy legislation [213,214,215]. By addressing these concerns holistically, the healthcare community can responsibly harness AI’s potential while maintaining rigorous patient protections.

## 6. Future Opportunities for AI in Biomedicine

### 6.1. AI in Emerging Biotechnologies

The rapid advancement of biotechnology has opened new avenues for improving healthcare outcomes, and artificial intelligence (AI) is poised to play a transformative role in this evolving landscape. Emerging biotechnologies, such as gene editing, synthetic biology, and nanotechnology, present unique challenges and opportunities that AI can help navigate.

**Figure 8:**
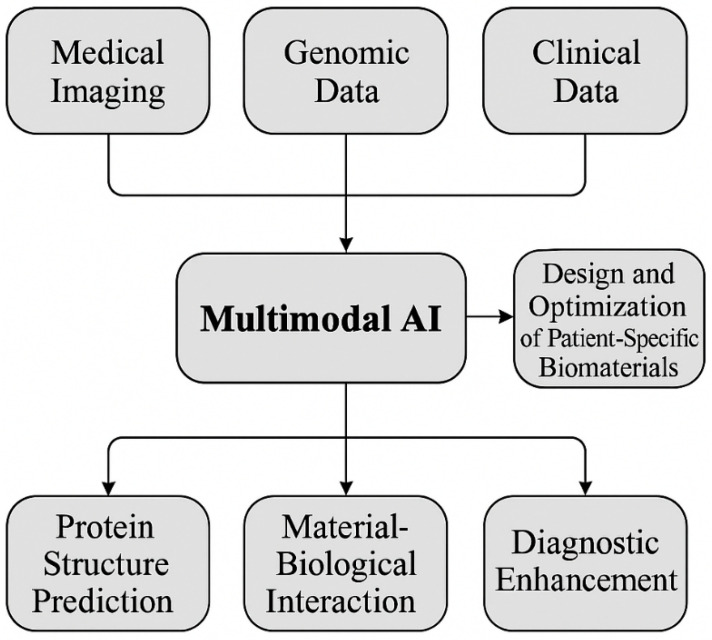
Schematic representation of multimodal AI in biomaterials science.

By integrating multimodal AI systems, researchers and clinicians can harness vast amounts of data from diverse sources to accelerate discovery, optimize processes, and personalize treatment strategies. Figure 8 illustrates the integration of various AI models (deep learning, machine learning, reinforcement learning, AlphaFold, and GANs) in biomaterials research. It highlights key data inputs, including medical imaging, genomic sequences, and material property datasets, feeding into AI-driven optimization processes [216]. The schematic also showcases AI applications in biomaterial design, tissue engineering, drug delivery, and regenerative medicine, while addressing challenges such as data bias, regulatory hurdles, and model interpretability. Moreover, these images serve as a creative prompt for discussions on ethical considerations and the integration of AI in clinical practice, encouraging stakeholders to envision a collaborative future between humans and intelligent systems. Ultimately, this figure underscores the need for continued research and development to harness AI’s full potential while addressing the challenges associated with its implementation in medical settings. Table 6 outlines the transformative future opportunities AI presents for biomedicine, highlighting how AI integration can improve precision, efficiency, and accessibility across various domains of healthcare and medical research [10,16,66,126,217,218,219,220,221,222].

The advent of CRISPR–Cas9 systems has transformed genetic manipulation, yet challenges persist in predicting editing outcomes and minimizing off-target effects. Artificial intelligence addresses these limitations through a sophisticated genomic analysis, enabling researchers to identify optimal target sequences and anticipate modification consequences with greater accuracy [223]. Machine learning models evaluate the sequence context and structural biology to forecast CRISPR activity, significantly improving the editing precision while reducing experimental iterations [224,225]. This synergy is particularly valuable for developing personalized genetic interventions, where AI algorithms analyze individual genomic profiles to design patient-specific editing strategies for monogenic disorders [226,227].

Artificial intelligence is re-engineering synthetic biology through the predictive modeling of complex biological systems. Deep learning architectures optimize microbial metabolic networks for enhanced bioproduction, identifying genetic modifications that maximize yields of therapeutic compounds and sustainable biomaterials [228,229]. Concurrently, AI-driven protein structure predictions enable the de novo design of functional biomolecules, creating novel enzymes and therapeutic proteins with tailored characteristics [230,231,232]. These computational tools are accelerating the development of next-generation biologics, from targeted drug delivery systems to innovative biologic therapies [233]. The intersection of AI and nanotechnology is pioneering new frontiers in precision medicine. Machine learning algorithms analyze structure–property relationships across nanomaterial libraries, enabling the rational design of particles with optimized characteristics for diagnostic and therapeutic applications [234,235]. Automated image analysis systems enhance nanomaterial characterization, rapidly assessing critical quality attributes while improving manufacturing consistency [236,237]. These AI-driven approaches are streamlining the translation of nanomedicines from bench to bedside, offering safer and more effective solutions for drug delivery and medical imaging [238]. Together, these technological convergences demonstrate how multimodal AI systems can overcome fundamental challenges across biotechnology domains, driving innovation in personalized therapeutics.

### 6.2. Integration of AI with 3D Bioprinting

The convergence of artificial intelligence with 3D bioprinting technologies is revolutionizing regenerative medicine by enabling the precise fabrication of customized biological constructs. AI-enhanced bioprinting systems leverage advanced computational modeling and data-driven optimization to improve the architectural complexity, functional performance, and clinical applicability of engineered tissues [239]. This synergistic integration addresses critical limitations in conventional bioprinting approaches while accelerating the development of patient-specific therapeutic solutions for tissue repair and organ regeneration.

The design phase of bioprinting is critical for achieving the desired structural and functional properties of bioprinted tissues. AI can significantly enhance this phase by optimizing the design process and enabling the creation of complex architectures that mimic natural tissues [240]. AI-driven computational design algorithms can analyze vast datasets of tissue characteristics, materials properties, and biological requirements to generate optimized designs for bioprinted constructs [241]. For example, machine learning models can assess the mechanical properties and biocompatibility of various biomaterials to recommend optimal combinations for specific applications [242]. By automating the design process, researchers can accelerate the development of bioprinted tissues that closely resemble their natural counterparts. AI can also be employed to predict the functional outcomes of bioprinted constructs based on their design parameters. By integrating multimodal data, such as mechanical testing results, biological assays, and imaging data, AI algorithms can identify correlations between design features and functional performance [243]. This predictive capability allows researchers to fine-tune their designs and optimize the functionality of bioprinted tissues before fabrication, reducing trial-and-error approaches [244]. The bioprinting process itself involves several parameters, including the printing speed, nozzle diameter, and material properties. AI can optimize these parameters to enhance the quality and reproducibility of bioprinted constructs [245]. Machine learning algorithms can analyze real-time data generated during the bioprinting process to identify optimal settings for various parameters. For instance, reinforcement learning techniques can be applied to adjust printing parameters dynamically based on feedback from the printing environment [246]. By optimizing these parameters, researchers can improve the fidelity of bioprinted tissues and reduce defects caused by variations in printing conditions [247]. AI can play a crucial role in quality control and assurance throughout the bioprinting process. By implementing computer vision techniques, AI systems can analyze images captured during printing to detect anomalies, such as misaligned layers or material inconsistencies [248]. Automated quality assurance processes can help ensure that bioprinted constructs meet the required specifications and standards, ultimately enhancing their reliability for clinical applications [249].

The post-processing phase of bioprinting involves additional steps to enhance the functionality and viability of bioprinted tissues. AI can aid in this phase by optimizing bioreactor conditions, assessing tissue maturation, and facilitating the functionalization of constructs [250]. AI-driven algorithms can analyze data from bioreactor systems to optimize culture conditions for bioprinted tissues. By integrating data on nutrient availability, mechanical stimulation, and cell behavior, AI can identify optimal parameters that promote tissue growth and functionality [251]. This approach allows for the development of bioprinted tissues that can better mimic the physiological conditions of native tissues [252]. AI can also assist in the functionalization of bioprinted constructs by predicting the effects of various biomolecular cues on cell behavior. By analyzing data on cell signaling pathways and molecular interactions, AI models can recommend specific growth factors or biomolecules to enhance tissue maturation and functionality [253]. This capability can lead to the development of bioprinted tissues with an improved integration into the host environment and enhanced therapeutic potential [254]. The integration of AI with 3D bioprinting represents a significant opportunity for advancing biomedicine. By enhancing the design, optimization, and functionalization of bioprinted constructs, AI can drive the innovation in tissue engineering and regenerative medicine. As researchers continue to explore the synergies between AI and bioprinting, the potential for creating patient-specific tissues and organs will expand, ultimately leading to improved healthcare outcomes and personalized treatment strategies.

### 6.3. Role of AI in Personalized Therapeutic Design

Contemporary medicine is undergoing a transformation through AI-enabled personalization, where treatment strategies are optimized using comprehensive patient profiles. By synthesizing multi-omics data, clinical histories, and lifestyle factors, machine learning algorithms reveal intricate biological patterns that inform tailored interventions [255]. Deep learning applications demonstrate a particular promise in correlating genomic variants with electronic health records, providing unprecedented insights into individual drug metabolism and therapeutic response patterns [256]. This data integration enables the identification of optimal treatment pathways while minimizing adverse effects through the precision targeting of disease mechanisms.

The convergence of AI with genomic medicine has revolutionized therapeutic personalization. Advanced algorithms analyze nucleotide sequences to detect pathogenic variants and predict medication efficacy, guiding clinicians in selecting genetically compatible treatments [257]. Beyond patient care, these computational tools accelerate novel drug development by identifying population-specific therapeutic targets through a large-scale genomic analysis [258]. AI-enhanced clinical decision support systems further refine treatment selection by dynamically incorporating emerging research, historical response data, and patient-specific biomarkers to generate real-time therapeutic recommendations [259,260]. This approach proves particularly valuable in drug repurposing, where machine learning mines vast pharmacological datasets to match existing compounds with new indications based on molecular signatures and patient subgroup characteristics [261,262,263].

The implementation of intelligent monitoring systems represents the next frontier in personalized care. Connected health technologies coupled with AI analytics enable the continuous assessment of therapeutic effectiveness through physiological tracking and medication adherence monitoring [264]. Predictive models process these real-time data streams alongside historical records to forecast treatment outcomes, allowing clinicians to preemptively modify regimens [265]. This dynamic approach ensures therapy remains optimized throughout the care continuum, adjusting for evolving patient responses while maintaining safety parameters [266]. As these technologies mature, they promise to establish a new standard of responsive, patient-centered medicine powered by intelligent data integration.

### 6.4. Potential for AI in Drug Discovery

Artificial intelligence is transforming pharmaceutical research by addressing key inefficiencies in traditional drug discovery pipelines. By processing multimodal biological data, AI accelerates target identification through the sophisticated analysis of genomic, proteomic, and metabolic networks, revealing novel disease-associated pathways [267,268,269]. Deep learning architectures excel at mining complex molecular datasets to pinpoint therapeutic targets, while predictive models assess the target druggability and potential clinical impact—significantly reducing early-stage attrition rates [270,271,272]. This data-driven approach uncovers promising interventions that conventional methods might overlook.

AI revolutionizes lead compound development through advanced computational chemistry techniques. Virtual screening algorithms rapidly evaluate millions of compounds for optimal target binding characteristics, with convolutional neural networks predicting pharmacological properties to prioritize candidates [273,274,275]. Generative AI models create novel molecular structures with tailored bioactivity profiles, using reinforcement learning to iteratively optimize parameters like potency and safety [276,277,278]. These innovations compress years of laboratory work into computational processes, enabling rational drug design at unprecedented scales.

The AI advantage extends through clinical translation, enhancing both efficiency and safety. Predictive toxicology models identify high-risk compounds before human trials, while machine learning optimizes trial protocols through patient stratification and adaptive design [279,280,281]. Real-time analytics during clinical studies enable dynamic protocol adjustments based on emerging efficacy and safety data [282,283]. As these technologies mature, AI-driven drug discovery promises to deliver more targeted therapies faster and at lower cost, ushering in a new era of precision pharmacotherapy.

### 6.5. Advantages and Limitations of Multimodal AI in Biomaterials Science

Multimodal AI has become an indispensable asset in biomaterials research, demonstrating exceptional capabilities in synthesizing heterogeneous biological data to advance material design and optimization. By simultaneously processing medical imaging, genomic profiles, and clinical parameters, these systems reveal complex structure–function relationships that inform biomaterial development. Sophisticated predictive algorithms, exemplified by protein-folding models like AlphaFold, enable the precise simulation of biological interactions, facilitating the rational engineering of tissue scaffolds and targeted drug carriers. Furthermore, AI empowers personalized biomaterial design through the machine learning analysis of patient-specific genetic markers and tissue characteristics, yielding implants and regenerative matrices with an enhanced biocompatibility and therapeutic performance.

However, several critical limitations currently constrain the broader implementation of AI in biomaterials science. Data-related challenges, including dataset incompleteness, quality inconsistencies, and inherent biases, compromise the model reliability and clinical generalizability. Translational hurdles also persist, with complex regulatory requirements and the absence of standardized validation protocols slowing clinical adoption. The opaque nature of deep learning architectures (“black box” problem) raises interpretability concerns, while substantial computational resource demands create accessibility barriers for many research institutions. Overcoming these obstacles will necessitate coordinated efforts to establish robust data-sharing infrastructures, develop explainable AI frameworks, and foster cross-disciplinary partnerships between computational scientists, material engineers, and clinical researchers.

Table 7 provides a comparative analysis of AI models in biomaterials science and highlights their diverse roles in biomaterial design, optimization, and clinical translation. Deep learning (DL) models, particularly convolutional neural networks (CNNs), have demonstrated superior pattern recognition capabilities, making them highly effective for imaging-based biomaterial characterization and tissue engineering applications [22,37,284,285,286,287,288]. However, their black-box nature and dependence on large datasets pose interpretability challenges. AlphaFold has revolutionized protein–material interaction modeling, allowing the precise prediction of bioactive scaffold designs, though its scope remains limited to protein-based biomaterials. Traditional machine learning (ML) models, such as Random Forest and Support Vector Machines (SVMs), offer computational efficiency and interpretability, making them suitable for small-to-medium-scale datasets, but they lack the deep feature extraction capabilities of DL models. Reinforcement learning (RL) enables self-optimizing biomaterial formulations, while generative adversarial networks (GANs) facilitate the design of novel biomaterials with enhanced properties, though training instability remains a concern. Hybrid AI models integrating DL, ML, and statistical methods offer a promising multimodal approach, leveraging diverse datasets for precision biomaterial development in tissue engineering, drug delivery, and regenerative medicine. Despite these advancements, the data quality, computational constraints, and regulatory challenges must be addressed to fully realize AI’s potential in biomaterials science.

### 6.6. Emerging Trends: Federated Learning, Digital Twins, and Clinical Translation

As AI continues to evolve, several emerging technologies are poised to significantly enhance its impact on biomedicine:

- Federated Learning for Privacy-Preserving AI: Federated learning is an emerging machine learning paradigm that enables model training across decentralized data sources without transferring sensitive patient data to a central server. This approach enhances data privacy and security while allowing AI models to learn from diverse healthcare datasets across institutions. It holds particular promise for collaborative biomaterials research and multi-center clinical studies, addressing data ownership concerns and complying with stringent privacy regulations.
- Integration with Digital Twins and 3D-Printed Biomaterials: Digital twins—virtual replicas of biological systems or patient-specific conditions—offer a powerful platform for simulating and optimizing biomaterial interactions, treatment responses, and implant integration. When combined with AI and 3D printing technologies, this integration enables the design of customized biomaterials tailored to individual patients’ anatomical and physiological profiles. AI-enhanced digital twins can predict outcomes of regenerative therapies or surgical implants, accelerating design iterations and improving therapeutic precision.
- Multimodal AI in Clinical Translation and Regulatory Approval: Multimodal AI systems that integrate clinical, molecular, and imaging data can streamline the clinical validation of new biomaterials and therapeutic devices. By providing more comprehensive and interpretable evidence of efficacy and safety, these systems can support regulatory submissions and facilitate approval pathways. Furthermore, explainable AI (XAI) techniques are increasingly being explored to meet transparency and accountability requirements in regulatory frameworks.

## 7. Conclusions

The incorporation of multimodal artificial intelligence into biomedical research and clinical practice marks a revolutionary shift in healthcare innovation, particularly for biomaterial development and advanced therapeutics. This analysis reveals several critical insights demonstrating AI’s capacity to redefine medical approaches. A primary advantage lies in AI’s proficiency at synthesizing heterogeneous biological data—encompassing molecular profiles, medical imaging, and clinical records—to generate holistic health assessments. Such an integrative analysis supports earlier disease detection, more precise prognostic evaluations, and customized treatment strategies. Within biomaterial science, an AI-driven computational design enables the fabrication of personalized constructs with optimized properties for tissue regeneration and implant applications. AI-enhanced diagnostic systems and precision treatment protocols are revolutionizing clinical care through the sophisticated analysis of complex medical datasets. These intelligent systems facilitate data-informed therapeutic decisions while enabling proactive healthcare through continuous monitoring and predictive capabilities. The convergence of AI with wearable biosensors and remote monitoring platforms creates unprecedented opportunities for real-time health tracking and patient-centered care models that emphasize prevention and early intervention. Nevertheless, significant implementation hurdles persist, including ethical dilemmas regarding data security, algorithmic transparency, and equitable access. Developing appropriate regulatory guidelines and ensuring scalable deployment across diverse healthcare settings remain essential for the responsible adoption of these transformative technologies.

Future Prospects for AI in Healthcare and Biomaterials

The horizon of multimodal AI in biomedical applications appears exceptionally bright, with emerging synergies between artificial intelligence and cutting-edge biotechnologies like CRISPR-based genome engineering and precision biomanufacturing. These convergences will enable the unprecedented customization of medical interventions, accounting for both genotypic and phenotypic patient variability. Particularly transformative is AI’s capacity to streamline pharmaceutical innovation through the rapid analysis of multidimensional biological data, identifying promising drug candidates and biomarkers with remarkable efficiency. Realizing this potential fully will demand sustained, cross-disciplinary partnerships that bridge computational science, clinical expertise, and fundamental research to translate technological advances into tangible global health benefits.

Final Thoughts on Multimodal AI’s Impact

Multimodal AI represents a revolutionary force in modern medicine, enabling the unprecedented integration of diverse biological datasets to drive precision healthcare solutions. This technological convergence facilitates a deeper understanding of disease mechanisms while creating opportunities for tailored therapeutic interventions. As research advances, these systems promise to transform biomaterial development and treatment paradigms, fostering more equitable and efficient healthcare delivery. Realizing this potential will require sustained interdisciplinary collaboration to address existing limitations and maximize the clinical impact of AI-driven innovations.

## Figures and Tables

**Table 1:** A comparative overview of the role of multimodal AI in biomaterials development.

Aspect	Traditional Biomaterials Development	Multimodal AI-Enhanced Biomaterials Development	Advantages of AI Integration	Ref
**Data Utilization**	Primarily relies on single-source data (e.g., biological assays)	Integrates diverse data sources (imaging, genomics, clinical data)	Provides holistic insights, improving biomaterial specificity and efficacy	[18]
**Material Design Approach**	Generalized designs based on population data or trial-and-error methods	Patient-specific designs based on individual health data	Enables precision and personalization in biomaterial properties	[19]
**Predictive Modeling**	Limited predictive capability, often requiring extensive experimentation	Advanced AI-driven modeling (e.g., AlphaFold for protein structures)	Reduces time and cost by predicting outcomes accurately before physical testing	[20]
**Optimization of Properties**	Based on empirical adjustments and physical testing iterations	AI analyzes complex relationships for optimal property tuning	Achieves targeted material properties efficiently for specific medical applications	[21]
**Interaction with Biological Systems**	Determined through iterative biocompatibility testing	AI predicts compatibility with biological systems using multi-omics data	Enhances biocompatibility and reduces adverse reactions	[22]
**Speed of Development**	Slower due to reliance on experimental validation	Accelerated through rapid AI-driven simulations and predictions	Shortens time-to-market for new biomaterials	[23]
**Application in Regenerative Medicine**	Limited personalization in grafts and scaffolds	AI customizes biomaterials to patient-specific regenerative needs	Promotes individualized tissue regeneration with higher success rates	[24]
**Scalability and Adaptability**	Challenging to scale personalized solutions	AI streamlines scalability by optimizing designs for diverse needs	Facilitates adaptable, scalable solutions for diverse patient populations	[25]
**Ethical and Regulatory Challenges**	Established guidelines for biomaterial safety	Emerging concerns over AI transparency, data privacy	Calls for updated regulatory frameworks and ethical standards	[26]

**Table 2:** AI-driven biomaterial design for tissue engineering.

Material Type	AI Tools/Methods Used	Biomedical Applications	Ref
**Polymeric Scaffolds**	Machine learning (ML) for structure–property prediction; Inverse design algorithms	Optimizing porosity, stiffness, and degradation rates for tissue-specific scaffolds (e.g., bone, neural, and musculoskeletal)	[35]
**Hydrogels**	Multimodal AI (imaging + molecular + clinical data fusion); Deep learning for ECM replication	Skin regeneration, wound healing, and extracellular matrix (ECM)-mimicking hydrogels	[37,38]
**Bone Scaffolds**	CT/MRI-based AI analysis (CNNs); Predictive modeling for porosity and mechanical strength	Patient-specific bone grafts with optimized pore architecture for osteogenesis	[36]
**Cartilage Scaffolds**	AI-driven cell-material interaction modeling; Predictive algorithms for chondrocyte behavior	Cartilage repair with enhanced cell adhesion and differentiation	[39,40]
**Vascular Grafts**	AI-based endothelial cell response prediction; Genetic algorithm-driven material optimization	Blood vessel regeneration with reduced thrombosis risk	[40]
**Immunocompatible Biomaterials**	Genomic data integration with ML; Immune response prediction models	Personalized implants with minimized rejection risks (e.g., skin grafts, and bone scaffolds)	[41,42]

**Table 3:** Comparative analysis of AI-driven diagnostic models in biomedicine.

AI Model	Medical Application	Accuracy (%)	Precision (%)	Recall (%)	Reference
CNN (ResNet-50)	Cancer Detection (Histopathology)	94.5	93.8	92.7	[50]
Deep Neural Network (DNN)	Cardiovascular Disease Prediction	89.2	87.5	88.3	[51]
Transformer-Based Model (BERT)	Medical Text Analysis (EHR Processing)	96.1	95.3	94.9	[52]
Generative Adversarial Networks (GANs) (CycleGAN)	Medical Image Enhancement	92.8	91.2	90.7	[53]
AlphaFold	Protein Structure Prediction	>92.4	N/A	N/A	[54]

N/A: Not Available.

**Table 4:** AI-optimized biomaterials for tissue engineering applications.

AI Model	Biomaterial Type	Application	Predicted Property (Accuracy %)	Biocompatibility (%)	Reference
ML-Based Model (SVM)	Hydrogel Scaffolds	Cartilage Regeneration	91.7	95.2	[24]
Deep Learning (CNN)	Nanocomposites	Bone Tissue Engineering	88.4	93.8	[36]
Bayesian Optimization	3D-Printed Biomaterials	Patient-Specific Implants	94.5	97.1	[22]
Reinforcement Learning	Bioactive Coatings	Antimicrobial Surfaces	90.3	92.6	[63]
AI-Guided GANs	Polymer-Based Biomaterials	Drug Delivery Systems	87.9	91.3	[64]

**Table 5:** Comparative overview of multimodal AI advancements and challenges in biomaterials science and personalized healthcare.

Aspect	Current State	Advancements with Multimodal AI	Challenges and Considerations	Future Implications
**Data Integration**	Data often remains siloed across imaging, genomics, and health records	AI combines diverse data sources, allowing holistic analysis for personalized material design	Ensuring data privacy, interoperability, and regulatory compliance	Enables comprehensive patient profiles for precision medicine
**Biomaterial Design**	Traditional biomaterials are designed based on generalized requirements	AI optimizes patient-specific biomaterials for drug delivery, tissue engineering, and regenerative applications	Complexity in validating AI-driven designs and achieving regulatory approvals	Patient-specific biomaterials that interact optimally with biological systems
**AI Tools (e.g., AlphaFold)**	Limited predictive accuracy for complex protein structures and interactions	High-accuracy prediction of protein folding and interactions	Algorithmic limitations in handling diverse biological contexts and data complexities	Accelerates biomaterial development tailored for biological compatibility
**Diagnostic Precision**	Diagnostic tools often rely on isolated data sources, limiting precision	AI-enhanced diagnostics integrate imaging, molecular, and clinical data for higher accuracy	Potential bias in algorithms and difficulty in validating AI-driven diagnostics	More accurate, personalized diagnoses supporting tailored treatments
**Wearable Health Monitoring**	Basic health tracking with limited data analysis capabilities	AI enhances real-time health monitoring for proactive interventions	Data privacy risks and lack of regulatory frameworks for wearable health data	Personalized health insights and early intervention possibilities
**Ethical and Regulatory Issues**	Emerging ethical standards; limited regulation for AI in healthcare	AI demands transparent and ethical algorithmic decisions for responsible healthcare use	Addressing algorithmic bias, data handling, and ensuring informed consent	Ethical and regulatory frameworks evolve, supporting AI integration in healthcare
**Potential for Personalized Medicine**	Limited precision and scalability in current treatments	AI enables tailored approaches, enhancing treatment efficacy and patient outcomes	Scalability, cost, and access barriers for AI-driven healthcare solutions	Transformative shift to data-driven, patient-centered healthcare systems

**Table 6:** Future opportunities for AI in biomedicine.

Opportunity Area	Current State	Future Potential with AI Integration	Impact on Biomedicine	Ref
**Personalized Medicine**	Limited to general population models	AI enables individualized treatments based on genetic, clinical, and lifestyle data	Enhances treatment efficacy and minimizes adverse reactions	[10]
**Drug Discovery and Development**	Time-intensive, costly with high attrition rates	AI accelerates drug discovery through predictive modeling and molecule screening	Reduces development time and cost, improving drug accessibility	[217]
**Predictive Diagnostics**	Diagnostics primarily based on isolated tests	Multimodal AI integrates diverse data for highly accurate, early diagnosis	Supports early intervention and improves prognosis	[16]
**Tissue Engineering and Regenerative Medicine**	Largely dependent on generalized biomaterials	AI designs patient-specific biomaterials for enhanced tissue compatibility	Improves patient outcomes and supports complex tissue repair	[66]
**Wearable Health Monitoring**	Limited to basic tracking (e.g., heart rate, steps)	AI-powered wearables analyze real-time data for personalized health insights	Facilitates proactive health management and early detection	[218]
**Genomic Analysis and Precision Genomics**	Analysis focused on specific genes or markers	AI analyzes entire genomes, identifying complex genetic interactions	Enables precise identification of genetic risk factors and mutations	[219]
**Remote and Telemedicine Applications**	Limited real-time patient analysis	AI enables real-time remote monitoring, diagnostic support, and patient triaging	Expands healthcare access, especially in underserved areas	[220]
**Ethical and Regulatory Frameworks**	Initial standards in place	AI-driven healthcare prompts the development of advanced ethical and regulatory models	Ensures responsible use, transparency, and patient trust	[126]
**AI-Augmented Surgical Procedures**	Limited AI assistance, primarily robotic arms	Future AI systems can assist in complex surgeries through real-time guidance	Enhances surgical precision and reduces risk of complications	[221]
**Health Data Management**	Fragmented data management across systems	AI consolidates data for seamless integration and patient care continuity	Improves care coordination and data-driven decision-making	[222]

**Table 7:** Comparative analysis of key AI models in biomaterials science.

AI Model	Application in Biomaterials Science	Strengths	Limitations	
**Deep Learning (DL)** (CNNs, RNNs)	Predicting biomaterial properties, image-based tissue analysis	High accuracy in feature extraction and pattern recognition	Requires large datasets; potential overfitting; lack of interpretability	[37]
**AlphaFold (Deep Learning-Based Structural Prediction)**	Protein-material interaction modeling, bioactive scaffold design	Highly accurate protein structure prediction; aids in rational biomaterial design	Computationally intensive; limited to protein-centric applications	[284]
**Machine Learning (ML) (Random Forest, SVM, Decision Trees)**	Biomaterial classification, mechanical property prediction	Efficient with small datasets; interpretable models	Performance depends on dataset quality; may lack deep feature extraction	[285]
**Reinforcement Learning (RL)**	Self-optimizing biomaterial formulations, drug delivery system design	Learns from trial-and-error; adaptive optimization	Requires extensive computational resources; long training times	[286]
**Natural Language Processing (NLP)**	Literature-based discovery of novel biomaterials	Automates knowledge extraction from vast scientific data	Limited understanding of contextual nuances in biomedical data	[287]
**Bayesian Networks**	Risk assessment in biomaterial biocompatibility and toxicity prediction	Handles uncertainty well; suitable for probabilistic modeling	Requires prior domain knowledge for accurate results	[22]
**Generative Adversarial Networks (GANs)**	Designing new biomaterials with optimized properties	Generates novel materials with desired characteristics	Training instability; potential for generating unrealistic structures	[288]
**Hybrid AI Models (Combining DL, ML, and Statistical Methods)**	Multimodal AI for patient-specific biomaterial optimization	Integrates diverse data sources for better decision-making	Complexity in integration; challenges in model validation	[22]
