# Correction: Parvin et al. Multimodal AI in Biomedicine: Pioneering the Future of Biomaterials, Diagnostics, and Personalized Healthcare. *Nanomaterials* 2025, *15*, 895

**DOI:** 10.3390/nano16010036

**Published:** 2025-12-25

**Authors:** Nargish Parvin, Sang Woo Joo, Jae Hak Jung, Tapas K. Mandal

**Affiliations:** 1 School of Mechanical Engineering, Yeungnam University, Gyeongsan 38541, Republic of Korea; nargish.parvin@gmail.com (N.P.); swjoo1@gmail.com (S.W.J.); 2 School of Chemical Engineering, Yeungnam University, Gyeongsan 38541, Republic of Korea

In the original publication [1], references 156 and 184 were retracted prior to this publication. Concerns were raised, and the authors would like to replace them with the following references:

156. Bouderhem, R. Shaping the future of AI in healthcare through ethics and governance. *Humanit. Soc. Sci. Commun.* **2024**, *11*, 416. https://doi.org/10.1057/s41599-024-02894-w.

184. Chen, Y.; Lehmann, C.U.; Malin, B. Digital Information Ecosystems in Modern Care Coordination and Patient Care Pathways and the Challenges and Opportunities for AI Solutions. *J. Med. Internet Res*. **2024**, *26*, e60258. https://doi.org/10.2196/60258.

The authors state that the scientific conclusions are unaffected. This correction was approved by the Academic Editor. The original publication has also been updated.
